# Supplementary material for: Predicting the comprehensive geospatial pattern of two ephedrine-type alkaloids for Ephedra sinica in Inner Mongolia
Source: PLoS One. 2023 Apr 21;18(4):e0283967. doi: 10.1371/journal.pone.0283967 (PMC10121062; doi:10.1371/journal.pone.0283967)
Supplement: Attachment — Submitted filename: PONE-D-22-06770_R1_ReviewerReport.pdf [file pone.0283967.s004.pdf]

Comments

Abstract: the period starting from L. 27 has several syntactical and grammatical errors, and it is too detailed about the optimization of model parameters. Consider replacing it with something like:

"First, we used the MaxEnt software to map suitable habitats for *E. sinica* in inner Mongolia, based on occurrence data and a set of variables related to climate, topography and human impact. MaxEnt parametrization was optimized by evaluating alternative combinations of feature classes and values of the regularization multiplier. Secondly, a geospatial quality model was fitted to relate E and PE content to the same environmental variables and to predict their spatial patterns across the study area. Outputs from the two models were finally combined to map areas predicted to host both suitable conditions for *E. sinica* and high alkaloid content. Our results indicate that high-quality E content of *E. sinica* was mainly distributed in the Horqin grassland…"

L.20: To improve clarity, consider replacing "is a type of traditional Chinese medicine that has a high medicinal value and large demands." with "is a shrubby plant widely used in traditional Chinese medicine due to high medicinal value and large demands".

L. 26: To improve clarity, consider adding "content" after "E and PE".

L. 44: Please replace "relying" with "relied" in the sentence "and the majority of these studies relying on species distribution models".

L. 45-47: I suggest reversing the construction of this sentence to improve clarity. For instance, you could write: "Various SDM algorithms, including CLIMEX, DOMAIN, genetic algorithm for rule set production (GARP), BIOMOD-2 and maximum entropy (MaxEnt), have been used to assess ecological requirements and predict habitat suitability for several medicinal plant species".

L. 53: Please remove "the" before "MaxEnt", here and throughout the manuscript unless you place words like "software", "algorithm", "model" after "MaxEnt".

L. 60: Please replace "influencing" with "influence".

L. 66: Please add "on" before "the suitable habitat" and before "the geospatial pattern".

L. 67-68: I suggest moving the phrase "We used a comprehensive quality model to determine the geospatial distribution of high-quality *E. sinica*" directly at the end of this paragraph (L.83), so that you start introducing the methods and scope of your study after having exemplified results from previous studies on medicinal plants. I also suggest adding "populations" after "*E. sinica*".

L. 78: Please replace "develop" with "assess/estimate/analyze".

L. 80-82: "they found that climatic factors wouldn't only be an indicator for identifying the potential distribution area, but also for forming some secondary metabolites such as baicalin" is a bit weird sentence…consider replacing it with something like "they found that climatic factors are useful indicators of both plants' potential distribution and production of some secondary metabolites such as baicalin".

L. 86: Please replace the comma after "colds and obesity[20]" with a semicolon or a full stop.

L. 100: Please replace “overharvested” with “overharvesting”.

L. 105: Add “geospatial” before “patterns” and add “these” before “two”.

L. 108: Consider replacing “the spatial pattern of two ephedrine-type alkaloids” with “the spatial patterns of E and PE content”.

L. 100: To improve clarity, consider replacing “habitat distribution and effective components” with “the distribution of suitable habitats for *E. sinica* and effective production of ephedrine-type alkaloids”.

L. 122: To improve syntactical correctness, consider replacing “…Ordos. While the western region is arid, with the Alxa desert.” with “…Ordos, while the western region is arid and includes the Alxa desert”.

L. 123: To improve syntactical correctness and clearness, consider replacing “…and sample sites with two ephedrine-type alkaloids were obtained through published researchers” with “…and sample sites with content data for the two target two ephedrine-type alkaloids were obtained through published literature”.

Table 1: Please replace “Isothermally” with “Isothermality”.

L.167-169: To improve clearness and readability, I would change the period structure to something like “By coupling a species distribution model with a geospatial quality model (GQM), we developed a comprehensive geospatial quality model (CGQM) to predict the geospatial pattern of two ephedrine-type alkaloids in *E. sinica* (Fig. 2).

Figure 2: “Envionment” instead of “Environmental” is still present in the “Geospatial quality model” panel; please fix it. Moreover, consider adding “implemented” before “methodological approaches” in the caption. Finally, to improve clearness of the Figure content, I also suggest:

- replacing “Spatial distribution of medicinal quality materials (GQM)” with “areas with high predicted alkaloid content”, also modifying the heading to “Geospatial Quality Model (GQM)”.

- replacing “High quality suitable habitat of major content” with “highly suitable areas with high predicted alkaloid content” in the “Comprehensive Geospatial Quality Model (CGQM)” panel.

L. 185: when writing “omission rates (E = 0.05)”, did you mean that you selected 0.05 as threshold value for omission rate thus considering only those models that did not exceed this value? Please clarify.

L. 189-191: This paragraph is poorly written. Consider adjusting to something like “Finally, the selected MaxEnt model was fitted using linear and threshold features (i.e. FC = lt), with RM = 3.5. The maximum number of iterations was set to 1000, logistic output format was set to logistic, and 10 replicated runs of cross-validation were used to reduce the uncertainty of the model.”

L. 195: Add the final “l” to “mode”.

L. 196: Please change “when it below 0.5 indicating…” to “with values lower than 0.5 indicating…”

L. 200-201: Consider moving this sentence about the split of predicted suitability into discrete classes to L. 193, so that it would be better connected to the description of MaxEnt output.

L.212-213: As in the previous version you stated that stepwise regression was used for model refinement (i.e. variable selection), consider changing this part to something like “We used SPSS (IBM SPSS Statistics 26; https://www.ibm.com/cn-zh/analytics/spss-statistics-software) to perform a stepwise regression for refining the initial GLM, thus maintaining only the informative predictors”.

L. 234: Following my previous comment, change “GLM-based” with “regression-based”.

L. 238-239: As the AIC value is not very informative by itself, you may remove it and change the sentence to “and AUC value of 0.960, indicating that the model performed well and was reliable in discriminating suitable areas for *E. sinica*”.

L.240-245: I suggest you to change “was” with “covered” when writing about the extent the different suitability classes spanned. Moreover, in explicating the extent of the different categories, avoid using XXX × YYY km^2: you should either use the form XXX × YYY km or directly enumerate the overall extent, such as ZZZ km^2. Please also change “‘High’ suitable habitat mainly distributed in…” with “highly suitable habitat was mainly distributed in…”.

L. 250-254: Here you state that the most influential variable was bio7, but Fig. 4a shows the partial response curve for bio17…please fix this.

L. 256: You should not start a sentence with “And” after a full stop. Use instead something like “Further”, “Moreover”, “Additionally”.

L. 257-260: I don’t think it is very informative to specify with such precision the range of values the partial response curves span. One reason is that this range could be influenced by the clamping procedure used within the MaxEnt software to extend the response curves beyond the range of the training values…for instance, how could the optimal value for ter be “1～1.9” if you state that it “ranged from 0.38 to 1”. I suggest you focus instead on the trends resulting from the curves (e.g. increasing suitability for *E. sinica* with higher values of ter and bio15) and on the range of values (not the precise values which are difficult to assess from the curves) corresponding to the highest predicted suitability and/or to abrupt changes in the trend of the focal curve.

L. 270: Please change “This result showed that the model had a remarkable effect,” with something like “This result suggests that the model explained a not negligible portion of the spatial variability in E content”. A model has no effect…the model you used just estimate the portion of variability in the response variable which could be causally related to the retained predictors…

L. 282: As for the previous comment, please replace “the model had a significant effect, and the result is reliable” with something like “the model has a noticeable explanatory power”.

L. 283: To improve clearness and syntactical correctness, please replace “and the spatial quality model result of PE in the study area shown in…” with something like “the projection of PE content across Inner Mongolia resulting from the corresponding geospatial quality model is shown in…”

L. 290-293: The wording here is a bit blurry…consider replacing with something like “Climate, and particularly temperature patterns, is apparently the main factor determining the accumulation of E

and PE in *E. sinica* samples because bio2 and bio8 emerged as the main variables influencing the spatial distribution of E and PE content”.

L. 298: To improve clearness and syntactical correctness, please replace “The high-quality E content in ISH was approximately 13.86 × 104 km2” with something like “the extent of ISH areas predicted to host high-quality E was approximately ZZZ km^2”.

L. 314-315: Consider replacing “and the AUC value was 0.960, indicating excellent performance in evaluating the accuracy of the results. Further, we used a GLM to…” with something like “and the AUC value was 0.960, indicating excellent discrimination performance. Further, we used a stepwise regression-based model to…”

L. 318: Add “these” before “two models”.

L. 320-321: You should better explicit how your results would help to “promote the sustainable use and appropriate conservation of *E. sinica* in Inner Mongolia”, for instance adding something like “by indicating which areas potentially host higher *E. sinica* populations with ephedrine-type alkaloid content so as to make its harvesting more efficient”. If you do this, you could then start the following sentence with “Our results will also make an important contribution to the enhancement of medicinal value and protection of biodiversity in Inner Mongolia”.

L. 326-327: Please remove this sentence as it is totally redundant with the previous one.

L. 333-334: The wording here is a bit awkward; consider changing to something like: “In addition, climate emerged as the main factor driving the distribution of *Rosa arabica* Crep. which frequently cooccurs with *Ephedra* species”.

L. 335: Please add “for *E. sinica*” after “habitat suitability”.

L. 339: “The temperature factor provides *E. sinica* an comfortable environment to grow up…” is a bit awkward sentence; consider replacing with something like “Annual temperature range contributes to determine the degree to which *E. sinica* populations of Inner Mongolia experience comfortable thermal conditions to grow up…”

L. 340: Please remove the word “factor” after precipitation…unless you’re referring to a specific precipitation-related variable it is uninformative.

L. 347: To avoid redundancy, consider this formulation: “…for the distribution of *E. sinica*, because it grows…”

L. 349-351: Here you state soil variables are also important, but this did not emerge from your MaxEnt model, so you should discuss why…for instance, you may hypothesize that soil properties are important drivers of *E. sinica* distribution at finer spatial scales than the regional scale of your research.

L. 352: “Secondary metabolite from plants is the unique sources…” is not so convincing. Pharmaceuticals, flavours etc. also derive from animal or mineral material. Please replace with “Secondary metabolites from plants are among the main sources…”

L. 357: Please correct to “Ephedrine-type alkaloids are the main secondary metabolites…”

L. 360: Please correct to “various mechanisms, which are regulated…”

L. 361: You should expand a bit about these “genetic strategies” to make the concept clearer to a reader not experienced of plants physiological regulatory mechanisms.

L. 365: Break the sentence here: “…temperature treatments[70]; for instance, the concentrations of ergine, ergonovine…”

L. 366: Please add something like “is maintained” or “is put” before “under short-term cold stress”.

L. 367: Add ‘range’ after ‘mean diurnal temperature’.

L. 367-369: As here you’re citing for the first time in the Discussion the two ephedrine-type alkaloids, you may use their extended names to help the reader recall what you’re talking about, then continuing with their abbreviations in the subsequent paragraphs.

L. 370: To ameliorate the wording, consider changing to something like “which was negatively correlated to PE content, so that the latter increases with lower temperature in the wettest season.”

L. 378: Please change ‘species’ to ‘populations/variety’ if you’re specifically referring to *E. sinica*.

L. 379: Consider changing “ecological environment” with “habitats”.

L. 384-388: Here you write “E. sincia” instead of “E. sinica”; please correct.

L. 384: Replace “have been” with “has been” as you’re referring to a singular subject, “*E. sinica*”.

L. 385: Add “populations” or “occurrence localities” after “existing *E. sinica*”.

L. 391: To avoid repeating “conservation” so many times, consider replacing “to achieve species conservation in recent years” with “to preserve plant species”.

L. 393-394: These two lines could be removed as you few lines later you specify the ISH areas which should be given restoration priority.

L. 396: Consider removing the “ISH covered ca. 42.18 × 104 km2” part, because repeating in the Discussion quantitative details you already reported in the Results is not so useful.

L. 399 and L. 405: You may remove the saxon genitive here as you never used it above when referring to *E. sinica*.

L. 425: Please change “need” to “needed” as here you’re referring to what you’ve done for your specific research.

L. 429: To improve the wording, consider changing to “…the medicinal parts of the target plant species”.

L. 430: “in future studies” is a bit redundant here, you may remove it.

L. 433: To improve the wording, consider changing to “..environmental variables, plant distribution as well as metabolite contents”.

L. 435-439: To improve the wording, consider changing to “…According to the chosen evaluation metrics, both models performed well, and they identified climate, human modifications and, secondarily, topography as key environmental factors affecting the distribution of *E. sinica* and the spatial pattern of alkaloids content. The fact that we used environmental predictors with

approximately 1 Km^2 resolution may facilitate extracting general guidelines on conservation and management of *E. sinica* in Inner Mongolia at a relatively fine spatial scale”.

L. 443: As for the start of the Discussion, I suggest using the extended name of E and PE here that you cite them for the first time in the Conclusions section.

L. 488: Add human modifications as a key predictor, as it emerged clearly within your updated Maxent model.

L. 449: Please replace “environmental variables” with “modelled habitat suitability”, as in its current form this sentence is unclear.
