# Supplementary material for: Predicting the comprehensive geospatial pattern of two ephedrine-type alkaloids for Ephedra sinica in Inner Mongolia
Source: PLoS One. 2023 Apr 21;18(4):e0283967. doi: 10.1371/journal.pone.0283967 (PMC10121062; doi:10.1371/journal.pone.0283967)
Supplement: Attachment — Submitted filename: Response to Reviewers.docx [file pone.0283967.s003.docx]

**Response Letter**

Journal requirements:

When submitting your revision, we need you to address these additional requirements.

**Response:** We thank Journal feedback and encouragement for revision. We thank Editor for his/her positive feedback and encouragement for revision.

1. Please ensure that your manuscript meets PLOS ONE's style requirements, including those for file naming. The PLOS ONE style templates can be found at

https://journals.plos.org/plosone/s/file?id=wjVg/PLOSOne_formatting_sample_main_body.pdf and

<https://journals.plos.org/plosone/s/file?id=ba62/PLOSOne_formatting_sample_title_authors_affiliations.pdf>".

**Response:** We have been ensured that our manuscript meets PLOS ONE's style requirements.

2. Thank you for stating the following financial disclosure:

“Funding for our research was provided by the National Natural Science Foundation of China (Grant No. 81072999).”

Please state what role the funders took in the study.  If the funders had no role, please state: ""The funders had no role in study design, data collection and analysis, decision to publish, or preparation of the manuscript.""

If this statement is not correct you must amend it as needed.

Please include this amended Role of Funder statement in your cover letter; we will change the online submission form on your behalf.

**Response:** Thanks for the suggestion, and we have been added the description “Funding for our research was provided by the National Natural Science Foundation of China (Grant No. 81072999). The funders had no role in study design, data collection and analysis, decision to publish, or preparation of the manuscript.” in our cover letter.

3. Thank you for stating the following in the Acknowledgments Section of your manuscript:

“We wish to express our gratitude to all the authors of this paper, all of whom provide useful feedback on an earlier version of the manuscript. Funding for our research was provided by the National Natural Science Foundation of China (Grant No. 81072999).”

We note that you have provided funding information that is not currently declared in your Funding Statement. However, funding information should not appear in the Acknowledgments section or other areas of your manuscript. We will only publish funding information present in the Funding Statement section of the online submission form.

Please remove any funding-related text from the manuscript and let us know how you would like to update your Funding Statement. Currently, your Funding Statement reads as follows:

“Funding for our research was provided by the National Natural Science Foundation of China (Grant No. 81072999).”

Please include your amended statements within your cover letter; we will change the online submission form on your behalf.

**Response:** Thanks for the suggestion, and we have been deleted the acknowledge in the manuscript and added the description “Funding for our research was provided by the National Natural Science Foundation of China (Grant No. 81072999). The funders had no role in study design, data collection and analysis, decision to publish, or preparation of the manuscript.” in our cover letter.

4. Thank you for stating the following in your Competing Interests section:

“The authors declare that they have no known competing financial interests or personal relationships that could have appeared to influence the work reported in this paper.”

Please complete your Competing Interests on the online submission form to state any Competing Interests. If you have no competing interests, please state ""The authors have declared that no competing interests exist."", as detailed online in our guide for authors at http://journals.plos.org/plosone/s/submit-now

This information should be included in your cover letter; we will change the online submission form on your behalf.

**Response:** The description of Competing Interests "The authors have declared that no competing interests exist" has been added in our cover letter.

5. Please amend either the abstract on the online submission form (via Edit Submission) or the abstract in the manuscript so that they are identical.

**Response:** We have been amended the online submission form to be consistent with the abstract in the manuscript.

6. We note that [Figures 1, 4, 6 & 7] in your submission contain [map/satellite] images which may be copyrighted. All PLOS content is published under the Creative Commons Attribution License (CC BY 4.0), which means that the manuscript, images, and Supporting Information files will be freely available online, and any third party is permitted to access, download, copy, distribute, and use these materials in any way, even commercially, with proper attribution. For these reasons, we cannot publish previously copyrighted maps or satellite images created using proprietary data, such as Google software (Google Maps, Street View, and Earth). For more information, see our copyright guidelines: http://journals.plos.org/plosone/s/licenses-and-copyright.

We require you to either (1) present written permission from the copyright holder to publish these figures specifically under the CC BY 4.0 license, or (2) remove the figures from your submission:

a. You may seek permission from the original copyright holder of [Figures 1, 4, 6 & 7] to publish the content specifically under the CC BY 4.0 license.

We recommend that you contact the original copyright holder with the Content Permission Form (http://journals.plos.org/plosone/s/file?id=7c09/content-permission-form.pdf) and the following text:

“I request permission for the open-access journal PLOS ONE to publish XXX under the Creative Commons Attribution License (CCAL) CC BY 4.0 (http://creativecommons.org/licenses/by/4.0/). Please be aware that this license allows unrestricted use and distribution, even commercially, by third parties. Please reply and provide explicit written permission to publish XXX under a CC BY license and complete the attached form.”

Please upload the completed Content Permission Form or other proof of granted permissions as an """"Other"""" file with your submission.

In the figure caption of the copyrighted figure, please include the following text: “Reprinted from [ref] under a CC BY license, with permission from [name of publisher], original copyright [original copyright year].”

b. If you are unable to obtain permission from the original copyright holder to publish these figures under the CC BY 4.0 license or if the copyright holder’s requirements are incompatible with the CC BY 4.0 license, please either i) remove the figure or ii) supply a replacement figure that complies with the CC BY 4.0 license. Please check copyright information on all replacement figures and update the figure caption with source information. If applicable, please specify in the figure caption text when a figure is similar but not identical to the original image and is therefore for illustrative purposes only.

The following resources for replacing copyrighted map figures may be helpful:

USGS National Map Viewer (public domain): http://viewer.nationalmap.gov/viewer/

The Gateway to Astronaut Photography of Earth (public domain): http://eol.jsc.nasa.gov/sseop/clickmap/

Maps at the CIA (public domain): https://www.cia.gov/library/publications/the-world-factbook/index.html and https://www.cia.gov/library/publications/cia-maps-publications/index.html

NASA Earth Observatory (public domain): http://earthobservatory.nasa.gov/

Landsat: http://landsat.visibleearth.nasa.gov/

USGS EROS (Earth Resources Observatory and Science (EROS) Center) (public domain): http://eros.usgs.gov/#

Natural Earth (public domain): <http://www.naturalearthdata.com/>.

**Response:** Thanks for the suggestion. We adjusted the order of the pictures. The data used in [Figures 1, 3, 5 & 6] were obtained from Natural Earth (http://www.naturalearthdata.com/) and WorldClim ([www.worldclim.org](http://www.worldclim.org)), and further processed using software ArcGIS 10.3 version. The data obtained from WorldClim can be used for mapping and spatial modeling (Yang et al., 2022 , West et al., 2016 , Elith et al., 2011). And we have descripted it in L. 145-146.

**Revision:** We have changed the description of figures:
Fig. 1. Study area and sampling sites distribution. The boundary was obtained from Natural Earth (http://www.naturalearthdata.com) and was further processed using software ArcGIS 10.3 version.
Fig. 3. Predicted potential habitats for E. sinica. This map was made in ArcGIS 10.3 using the resulting raster produced by MaxEnt. The original boundary was obtained from Natural Earth (http://www.naturalearthdata.com).
Fig. 5. a. E content across Inner Mongolia; b. E content in important suitable habitats for E. sinica This map was made in ArcGIS 10.3 using the resulting rasters produced by geospatial quality mode and distribution model. The original boundary was obtained from Natural Earth (http://www.naturalearthdata.com).
Fig. 6. a. PE content across Inner Mongolia; b. PE content in important suitable habitats for E. sinica. This map was made in ArcGIS 10.3 using the resulting rasters produced by geospatial quality mode and distribution model. The original boundary was obtained from Natural Earth (http://www.naturalearthdata.com).

**References:**
Yang, J.T., et al., Potential geographic distribution of relict plant Pteroceltis tatarinowii in China under climate change scenarios. Plos One, 2022. 17(4).
West, A.M., et al., Field validation of an invasive species Maxent model. Ecological Informatics, 2016. 36: p. 126-134.
Elith, J., et al., A statistical explanation of MaxEnt for ecologists. Diversity and Distributions, 2011. 17(1): p. 43-57.

Reviewer #1: The MS Predicting the comprehensive geospatial pattern of two ephedrine-type alkaloids for E.sinicain Inner Mongolia. Even though plant conservation is an important concern in a world that is changing, this MS cannot improve the conservation strategies for the researched plant in its current state. This MS is now a modeling exercise rather than a scientific study.

This MS cannot be approved for publishing unless substantial research and recommendations for habitat protection and conservation planning are included.

As stated by the context, indiscriminate destruction and grassland degradation pose major risks to species, and merely forecasting the species' probable range is insufficient, as these are in dire need of conservation.

It is beneficial to have distribution maps and predicted geographic trends, but it is unclear, and the author fails to address how to apply this knowledge for species conservation.

One of the ideas is that as authors are eager to predict the distribution of species, one of the ideas is that they evaluate if ensemble modelling is useful for optimising the predictive performance of a species distribution model in this instance.

Furthermore, authors should additionally incorporate other limiting factors, such as human pressure (e.g., human impact index) and other habitat disturbances that might further constrain the niche of the species into the model. The authors should study various local-scale techniques, such as PAB (propagule pressure, abiotic and biotic). Authors should utilize future possibilities as well. There is the potential for enhancing the models using variables such as "future land use and cover change scenarios."

**Response:** We thank Reviewer 1 for his/her thorough review, positive feedback, and constructive comments. For the question of “research and recommendations for habitat protection and conservation planning should be included.”, we agree with it and added the description of the plant’s protection and conservation. For the question of “authors should additionally incorporate other limiting factors, such as human pressure (e.g., human impact index) and other habitat disturbances that might further constrain the niche of the species into the model.”, the human activity variables have been add in our research, which including human footprint, population density and global human modification of terrestrial system (L. 169-171) (Table 1). And the human activity is an important factor for distribution of E. sinica.

**Revision:**

(1) “**4.2. Conservation strategies for Ephedra sinica**
 As a result of rapidly changing climate and human-caused ecological destruction, the distribution of E. sinica in Inner Mongolia has shifted from widespread to fragmented in recent decades (Gonzalez-Juarez et al., 2020, Yin et al., 2016). Meanwhile, because of the high medicinal value of E. sincia, which have been widely used in both traditional Chinese medicine and Western medicine, existing E. sincia and potential suitable areas are critical for the future protection and reforestation of E. sincia. We propose the following conservation management strategies for E. sincia in Inner Mongolia based on our findings.
 Conservation methods such as in situ conservation, near-field conservation, ex situ conservation, and germplasm conservation have been gradually applied to achieve species conservation in recent years (Hu et al., 2020). In situ protection should be strengthened in regions where wild E. sinica exits, and protection-relevant policies and measures to prohibit the destruction of wild E. sinica can be issued. Furthermore, reasonable restoration project planning should be encouraged in potential suitable habitat areas where we predicted. The majority of important suitable habitat for E. sinica in our study occurred in Horqin grassland, Wulanchabu grassland, Ordos grassland and Ulan Butong grassland. ISH covered ca. 42.18 × 104 km2. These regions, accounting for 35% of Inner Mongolia. should be given top priority for restoration. Introduction and cultivation of wild E. sincia are important ways to restore this plant, and the government can increase farmers' enthusiasm for planting E. sinica through policies and economic incentives. Meanwhile, E. sinica’s cultivation should be based on mimicking the wild cultivation mode (Meng et al., 2018). Taking other species into account when conducting Ephedra resource protection, such as Haloxylon ammodendron, Hippophae rhamnoides Linn, and Glycyrrhiza uralensis Fisch, could reduce environmental vulnerability and aid in the survival of E. sinica (He et al., 2021).” (L. 398-425).

(2) “**Human activity variables** included human footprint (Venter et al., 2016), population density (Center for International Earth Science Information Network - CIESIN - Columbia University, 2018) and global human modification of terrestrial system (Kennedy et al., 2020), which were derived from the Socioeconomic Data and Applications Center (http://sedac.ciesin.columbia.edu).”

| **Table 1**  Environmental variables used or not used in model, along with the respective relative contribution scores from the fitted Maxent model | | | |  |
| --- | --- | --- | --- | --- |
| Variable type | Code(Unit) | Description | Variables used in modeling | Contribution (%) |
| Climatic variables | bio1(℃) | Annual mean air temperature | √ | 2.1 |
|  | bio2(℃) | Mean diurnal temperature range (max. temp – min. temp) | √ | 0.8 |
|  | bio3 | Isothermally (Bio2/Bio7) × 100 | √ | 4.3 |
|  | bio4(℃) | Temperature seasonality |  |  |
|  | bio5(℃) | Max temperature of warmest month |  |  |
|  | bio6(℃) | Min temperature of coldest month |  |  |
|  | bio7(℃) | Temperature annual range | √ | 26.6 |
|  | bio8(℃) | Mean temperature of wettest quarter |  |  |
|  | bio9(℃) | Mean temperature of driest quarter |  |  |
|  | bio10(℃) | Mean temperature of warmest quarter |  |  |
|  | bio11(℃) | Mean temperature of coldest quarter |  |  |
|  | bio12(mm) | Annual precipitation | √ | 8.4 |
|  | bio13(mm) | Precipitation of wettest month |  |  |
|  | bio14(mm) | Precipitation of driest month |  |  |
|  | bio15(%) | Coefficient of variation of precipitation | √ | 19.3 |
|  | bio16(mm) | Precipitation of wettest quarter |  |  |
|  | bio17(mm) | Precipitation of the driest quarter |  |  |
|  | bio18(mm) | Precipitation of warmest quarter |  |  |
|  | bio19(mm) | Precipitation of coldest quarter |  |  |
|  | srad(kJ·m^-2^·d^-1^) | Solar radiation | √ | 10.9 |
|  | vapr(hPa) | Vapour pressure | √ | 0.6 |
| Soil variables | soil | Soil type | √ | 0.3 |
|  | clay1 | Topsoil Clay Fraction (0 - 30cm) |  |  |
|  | clay2 | Subsoil Clay Fraction (30 - 100cm) |  |  |
|  | sand1 | Topsoil Sand Fraction (0 - 30cm) | √ | 0.7 |
|  | sand2 | Subsoil Sand Fraction (30 - 100cm) |  |  |
|  | sq1 | Nutrient availability | √ | 0.3 |
|  | sq2 | Nutrient retention capacity |  |  |
|  | sq3 | Rooting conditions | √ | 1.1 |
|  | sq4 | Oxygen availability to roots |  |  |
|  | sq5 | Excess salts | √ | 1.1 |
|  | sq6 | Toxicity | √ | 0.2 |
|  | sq7 | Workability (constraining field management) |  |  |
| Topographical variables | ele(m) | Elevation above sea level | √ | 0.2 |
|  | slop(%) | Slope |  |  |
|  | asp(degrees) | Aspect | √ | 0.1 |
| Human activity variables | hf | Human Footprint |  |  |
|  | den | Population Density |  |  |
|  | ter | Global Human Modification of Terrestrial Systems | √ | 23 |

Reviewer #2: Dear Authors,
I’ve been glad to review your manuscript entitled “Predicting the comprehensive geospatial pattern of two ephedrine-type alkaloids for E. sinica in Inner Mongolia”. I think the idea of coupling the predictions of habitat suitability for E. sinica resulting from Maxent with a spatial interpolation model focusing on the content of its two ephedrine-type metabolites may indeed allow you to provide guidance for a smart and conservative utilization of this plant. However, I have some major concerns in the way the Maxent model was fitted and evaluated, in the way you described the model building steps in your Methods section, as well as about some parts of the Results.
Pease find below my major comments, along with a set of minor comments aiming to aid you improving the clearness and conciseness of your manuscript.
Sincerely,
Francesco Cerasoli
**Response:** We thank Reviewer 2 for his/her thorough review, positive feedback, and constructive comments.

Major comments
Introduction
L. 78: “High-quality species niches are different from species niches” is an incorrect formulation in my opinion. The niche of a species, intended as the Hutchinsonian multivariate fundamental niche, is one. What may differ among the different areas where a species is present is the degree to which the values of the abiotic factors characterizing those areas are close to the optimal range of values along the relevant axes of this multivariate niche space. Thus, I suggest replacing your statement with something like “areas hosting suitable conditions for high-quality populations of a pharmacological plant species (i.e. populations whose individuals show high content of the plant’s medicinal components) may not completely match areas where the species actually occurs”.

**Response:** Thanks for the careful review. We have been replaced it (L. 78-79).

**Revision:** “areas hosting suitable conditions for high-quality populations of a pharmacological plant species (i.e. populations whose individuals show high content of the plant’s medicinal components) may not completely match areas where the species actually occurs”

Methods
1. You should specify the resolution (e.g. 30 arc-seconds) of the different sets of environmental variables you initially considered.

**Response:** We apologize for missing the exact description of resolution, and a description of the data resolution have been added (L. 170-171).

**Revision:** "All environmental variables had a 30-s (approximately 1 km2) spatial resolution."

2. You should specify which link function (I suppose Gaussian) you used to model the content of E and PE as a function the variables selected in the stepwise process.

**Response:** We used the Gaussian type for link function, and have been descripted it at L. 228.

**Revision:** ”In the GLM, the linear predictor (LP) is combined with the predictor variables X_p(p=1,2,…,j) where α is the intercept, β is the vector of the regression coefficients, μ=E(Y) denotes the conditional expectation of Y given X= x, and "g()" denotes the link function**; here we chose the Gaussian type**.”

3. You relied on the high AUC value your Maxent model attained on the test data to state that the corresponding model predictions are highly reliable. Nonetheless, lots of papers on ENMs/SDMs in recent years have shown that high AUC scores could be also artifacts relating to spatial autocorrelation between the training and test datasets, as well as depending on the relative extent of occurrence of the target species across the study area (see for instance Jiménez-Valverde et al., 2013; Roberts et al., 2017; Cerasoli et al., 2022). As you state that you used the default splitting criterium of the Maxent program (i.e. “Crossvalidate”), you could have ended up with test data being spatially (and likely environmentally) close to the training ones, which in turn could have inflated the AUC score.
Thus, to make your model even more reliable, I suggest you either:
o Use the ‘enmeval’ R package (Kass et al., 2022). This would allow you to define a spatially and/or environmentally clustered cross-validation structure which would reduce the risk of spatial autocorrelation between training and test data, and then to find the combination of parameters (i.e. value regularization multiplier, feature classes) which most reduces the difference between the AUC computed on training data and that computed on test data (i.e. reducing model overfitting).
o Add an additional model evaluation step using the Boyce index, which is more suited for presence-background algorithms as Maxent (Hirzel et al., 2006; Cerasoli et al., 2021).

**Response:** We agree with the reviewer that “high AUC scores could be also artifacts relating to spatial autocorrelation between the training and test datasets”. We use the “kuenm” R package for picking the most appropriate parameters, and used (i) omission rates (E = 0.05), which represent the proportion of incorrectly predicted test records by the model, (ii) Delta Akaike Information Criterion (dAIC <2) to determine the final combination of parameters.

“kuenm” R package a set of tools for performing detailed development of ecological niche models using the platform Maxent in a reproducible way. The fitted MaxEnt Model in our study was built with the selected parameters; the combination of the FC was lt, and the RM was 3.5. The selected model presented an omission rate of 0.05, dAIC of 0, and AUC value was 0.960.

**Revision:** " **The feature classes (FC) and regularization multiplier (RM) influence the accuracy of the MaxEnt model** (Phillips and Dudik, 2008, Cerasoli et al., 2022). Thus, we used the **‘kuenm’ package in R software** to pick the most appropriate parameters for MaxEnt simulating the potential distribution of E. sinica (Cobos et al., 2019). 75% of the occurrence data were randomly selected to fit the model, and the remaining was used to test the model. In total, 248 candidate models were tested by combining RM values ranging from 0.5 to 4.0 (increments of 0.5, total 8 values) and all 31 combinations of five FCs setting (l, q, p, t, h; where l = linear, q = quadratic h = hinge, p = product and t = threshold) (Cobos et al., 2019). Model selection was based on:(i) omission rates (E = 0.05), which represent the proportion of incorrectly predicted test records by the model, (ii) Delta Akaike Information Criterion (dAIC <2), indicating models with the best trade-offs between data fitting and complexity (Escobar et al., 2018). The dAIC was the difference between a particular model’s AIC and the lowest AIC of all candidate models (Casas et al., 2022). Finally, MaxEnt Model was built with the selected parameters; the combination of the **FC was lt,** and the **RM was 3.5.** And 10 replicated runs of cross-validation were used to reduce the uncertainty of the model.”

**References:**
CASAS E, MARTIN-GARCIA L, HERNANDEZ-LEAL P, et al. 2022. Species Distribution Models at Regional Scale: Cymodocea nodosa Seagrasses. Remote Sensing [J], 14. http://doi.org/10.3390/rs14174334.
CERASOLI F, D'ALESSANDRO P, BIONDI M 2022. Fine-Tuned Ecological Niche Models Unveil Climatic Suitability and Association with Vegetation Groups for Selected Chaetocnema Species in South Africa (Coleoptera: Chrysomelidae). Diversity-Basel [J], 14. http://doi.org/10.3390/d14020100.
COBOS M E, PETERSON A T, BARVE N, et al. 2019. kuenm: an R package for detailed development of ecological niche models using Maxent. Peerj [J], 7. <http://doi.org/10.7717/peerj.6281>.
CASAS E, MARTIN-GARCIA L, HERNANDEZ-LEAL P, et al. 2022. Species Distribution Models at Regional Scale: Cymodocea nodosa Seagrasses. Remote Sensing [J], 14. <http://doi.org/10.3390/rs14174334>.
ESCOBAR L E, QIAO H, CABELLO J, et al. 2018. Ecological niche modeling re-examined: A case study with the Darwin's fox. Ecology and Evolution [J], 8: 4757-4770. http://doi.org/10.1002/ece3.4014.
PHILLIPS S J, DUDIK M 2008. Modeling of species distributions with Maxent: new extensions and a comprehensive evaluation. Ecography [J], 31: 161-175. http://doi.org/10.1111/j.0906-7590.2008.5203.x.

Results
L. 261: “the linear relationship between the dependent variable and independent variable was obvious”…which independent variable? From the β parameters it seems that aspect variations have a poor influence on the E content (βaspect = 0.001). The same “criticism” applies to the Discussion section, L. 353-356, where you highlight the presumed importance of topographic factors on E content.

**Response:** We apologize for incorrect description that aspect variations have an important influence on the E content. The aspect factor has a poor influence on the E content indeed, thus we have corrected descriptions (L. 294-296, 393-397).
**Revision:** “This result showed that the model had a remarkable effect, and the key variable of E content accumulation was mean diurnal temperature range. The spatial distribution of the E content in the different regions is shown in Fig. 5a.” and “mean diurnal temperature was the key variable for the generation of E, and it is positively correlated with the change of E content. When the temperature rises, E content will increase. The most important factor for the production of PE was the mean temperature of the wettest quarter, and its change causes the reverse change of PE, that is, when it decreases, PE increases.”

L. 330-332, and L. 336-338: According to the relative contribution scores you reported, precipitation seasonality (bio15) was the third most influential variable, not precipitation of the driest quarter (bio17). Please correct the corresponding parts of the Discussion.

**Response:** We have been fixed the description of bio15 throughout the manuscript. Due to we added the human activity variables：human footprint (Venter et al., 2016), population density (Center for International Earth Science Information Network - CIESIN - Columbia University, 2018) and global human modification of terrestrial system (Kennedy et al., 2020), which were derived from the Socioeconomic Data and Applications Center (<http://sedac.ciesin.columbia.edu>) (Table 1). Therefore, important factors changed. (L. 166-170). Meanwhile, we have revised the discussion part of important variables (L. 349-350, 362-365)

**Revision:**

2.2.	Environmental data **“**the internal jackknife test of factor importance showed that bio7 (annual temperature range, accounting for 26.6%) was the most important influencing factor, followed by ter (global human modification of terrestrial system, 23%), bio15 (Coefficient of variation of precipitation, 19.3%), srad (solar radiation, 10.9%) and bio12 (annual precipitation, 8.4%) (Table 1), and the cumulative contribution reached 88.2%”

4.1. Effects of environmental variables on Ephedra sinica
“Similar to previous studies, our results indicated that climate-related predictors were the main variables affecting habitat suitability. The major climate variables predicting the distribution of E. sinica were temperature annual range (26.6%), precipitation seasonality (19.3%), solar radiation (10.9%) and annual precipitation (8.4%).” and “. Furthermore, global human modification of terrestrial systems (23%) is an important variable for the distribution of E. sinica, because E. sinica grows primarily in grasslands, where human farming and animal husbandry activities and cultivation behavior change the land cover state.”

| **Table 1**  Environmental variables used or not used in model, along with the respective relative contribution scores from the fitted Maxent model | | | |  |
| --- | --- | --- | --- | --- |
| Variable type | Code(Unit) | Description | Variables used in modeling | Contribution (%) |
| Climatic variables | bio1(℃) | Annual mean air temperature | √ | 2.1 |
|  | bio2(℃) | Mean diurnal temperature range (max. temp – min. temp) | √ | 0.8 |
|  | bio3 | Isothermally (Bio2/Bio7) × 100 | √ | 4.3 |
|  | bio4(℃) | Temperature seasonality |  |  |
|  | bio5(℃) | Max temperature of warmest month |  |  |
|  | bio6(℃) | Min temperature of coldest month |  |  |
|  | bio7(℃) | Temperature annual range | √ | 26.6 |
|  | bio8(℃) | Mean temperature of wettest quarter |  |  |
|  | bio9(℃) | Mean temperature of driest quarter |  |  |
|  | bio10(℃) | Mean temperature of warmest quarter |  |  |
|  | bio11(℃) | Mean temperature of coldest quarter |  |  |
|  | bio12(mm) | Annual precipitation | √ | 8.4 |
|  | bio13(mm) | Precipitation of wettest month |  |  |
|  | bio14(mm) | Precipitation of driest month |  |  |
|  | bio15(%) | Coefficient of variation of precipitation | √ | 19.3 |
|  | bio16(mm) | Precipitation of wettest quarter |  |  |
|  | bio17(mm) | Precipitation of the driest quarter |  |  |
|  | bio18(mm) | Precipitation of warmest quarter |  |  |
|  | bio19(mm) | Precipitation of coldest quarter |  |  |
|  | srad(kJ·m^-2^·d^-1^) | Solar radiation | √ | 10.9 |
|  | vapr(hPa) | Vapour pressure | √ | 0.6 |
| Soil variables | soil | Soil type | √ | 0.3 |
|  | clay1 | Topsoil Clay Fraction (0 - 30cm) |  |  |
|  | clay2 | Subsoil Clay Fraction (30 - 100cm) |  |  |
|  | sand1 | Topsoil Sand Fraction (0 - 30cm) | √ | 0.7 |
|  | sand2 | Subsoil Sand Fraction (30 - 100cm) |  |  |
|  | sq1 | Nutrient availability | √ | 0.3 |
|  | sq2 | Nutrient retention capacity |  |  |
|  | sq3 | Rooting conditions | √ | 1.1 |
|  | sq4 | Oxygen availability to roots |  |  |
|  | sq5 | Excess salts | √ | 1.1 |
|  | sq6 | Toxicity | √ | 0.2 |
|  | sq7 | Workability (constraining field management) |  |  |
| Topographical variables | ele(m) | Elevation above sea level | √ | 0.2 |
|  | slop(%) | Slope |  |  |
|  | asp(degrees) | Aspect | √ | 0.1 |
| Human activity variables | hf | Human Footprint |  |  |
|  | den | Population Density |  |  |
|  | ter | Global Human Modification of Terrestrial Systems | √ | 23 |

**References added:**
KENNEDY C M, OAKLEAF J R, THEOBALD D M, et al. 2020. Global Human Modification of Terrestrial Systems [M]. NASA Socioeconomic Data and Applications Center (SEDAC); Palisades, New York.
VENTER O, SANDERSON E W, MAGRACH A, et al. 2016. Sixteen Years of Change in the Global Terrestrial Human Footprint and Implications for Biodiversity Conservation. Nature Communications [J]: 12558.

Minor (Text-related) comments
Title: You should report the extended binomial name Ephedra sinica in the title.

**Response:** We have corrected the title to “Predicting the comprehensive geospatial pattern of two ephedrine-type alkaloids for *Ephedra sinica* in Inner Mongolia”.

L. 29: I would say “highly suitable areas for E. sinica” rather than “high-quality suitable area of E. sinica”.

**Response:** We have replaced it with “highly suitable areas for *E. sinica*” (L. 35).

L. 30: I would add “these” before “Two ephedrine-type alkaloids”.

**Response:** We have been added it (L. 36).

L. 52: “Ecological environment” is a bit weird definition, consider replacing with “natural environments”. Here and in the other parts of the manuscript where you used this expression.

**Response:** Agree, we deleted the “Ecological environment”, and replaced the whole sentence. (L.54-55).

**Revision:** “many Chinese medicinal plants have declined in quantity and quality as a result of global climate change and human activities, with some on the verge of extinction.”

L. 56: “and important environmental factors” is maybe too generic; consider replacing with something like “and to assess the factors shaping environmental suitability for these latter”.

**Response:** we deleted the “and important environmental factors”, and replaced the whole sentence. (L.56-58).

**Revision:** “Previous research on potential suitable habitats of medicinal plants has aided TCM protection, and the majority of these studies relying on species distribution models (SDMs) to predict medicinal plants distribution.”

L. 63: Replace “Pepnia rockii” with “Peonia rockii”.

**Response:** We have been corrected it (L. 65).

L. 66: In the Ecological Niche Modelling (ENM) literature, the “potential” adjective is mostly associated to the noun “distribution”. Indeed, ENM algorithms usually take advantage of occurrence data, usually depicting only a portion of the realized niche of the target species, to estimate the potential distribution of the species across the landscape (Peterson & Soberón, 2012). Thus, I suggest replacing “potential ecological niches” with “potential distribution”.

**Response:** We have been corrected it (L.67).

**Revision:** “Gaikwad et al. (2011) used the MaxEnt to predict the potential distribution of 431 indigenous medicinal plant species in Australia based on bioclimatic variables.”

L. 92: I suggest replacing “this plant results in” with “the medicinal components of this plant favour”.

**Response:** We have replaced it (L. 102).

**Revision:**” the medicinal components of this plant favor sweating, alleviating colds, promoting lung health, relieving asthma, and detumescence.”

L. 101: I suggest replacing “etc.” with “along with other beneficial effects”.

**Response:** We have replaced it (L. 110).

**Revision:** “E is an effective component in providing analgesia services (Wang, 2019), regulating blood pressure (Houssiau et al., 2004), stimulating the central nervous system (Yu et al., 2009), relaxing bronchial smooth muscles (Lin et al., 2016), along with other beneficial effects.”

L. 104: “ephedrine-type alkaloids are affected by different species and spatial locations” is not so clear; you may change with something like “ephedrine-type alkaloids differ in content and quality among species and geographical locations”.

**Response:** We replaced it with “ephedrine-type alkaloids differ in content and quality among species and geographical locations” (L. 112-113)

**Revision:**” Previous studies have shown that ephedrine-type alkaloids differ in content and quality among species and geographical locations.”

L. 107: “ecological environmental improvements” is a bit weird formulation. Consider replacing by “ecological enhancement”.

**Response:** It has been corrected (L. 116).

**Revision:**” Windbreaks, sand fixation, soil erosion prevention, grassland protection and ecological enhancement on sandy land are all important uses for E. sinica.”

L. 110: Add blank spaces between the single words in “useEand”.

**Response:** It has been corrected, and we change the whole sentence with “our goal was to examine the comprehensive geospatial pattern using E and PE as quality indicators for evaluation and utilization of E. sinica.” (L. 120-121)

L. 111: Consider replacing “of E. sinica to analyze…” with “of E. sinica, by analyzing…”.

**Response:** We change the whole sentence (L. 120-121).

**Revision:**” our goal was to useEand PE as quality indicators for the evaluation and utilization of E. sinica to analyze the comprehensive geospatial pattern of E and PE contents” with “our goal was to examine the comprehensive geospatial pattern using E and PE as quality indicators for evaluation and utilization of E. sinica.”

L. 115: Add “potential” before “distribution”.

**Response:** We added it （L. 122）

L. 123: I think it is better to use the extended binomial name of the target species within the sections’ headings and sub-headings.

**Response:** We have been used “Ephedra sinica” in the section’ heading and sub-heading throughout the manuscript (L. 130, 256, 307, 336, 396, 419).

L. 124: Replace “and E. sinica” with “this plant/species”.

**Response:** We have changed the whole sentence (L. 131-132).

**Revision:** “E. sinica is primarily distributed in Inner Mongolia Autonomous Region of China (37°24′~53°23′N, 97°12′~126°04′E) (Herzschuh et al., 2003).”

L. 131-132: To avoid repeating the word “grassland” too many times, you could simply state “The main grassland areas of Inner Mongolia are Hulun Buir, Uragai, Xilingol…”.

**Response:** We have replaced the whole sentence with “The grasslands are mostly distributed in eastern and central Inner Mongolia, including Hulun Buir, Uragai, Xilingol, Horqin, Ulan Butong, Wulanchabu and Ordos.” (L. 135-137).

L. 138: “improve the credibility of the model.” is too generic, you could state “to reduce the potential detrimental effect of spatial autocorrelation in the training dataset” or simply “to avoid redundancy in the information used to fit the model”.

**Response:** We have been replaced it with “to reduce the potential detrimental effect of spatial autocorrelation in the training dataset” (L. 142-143).

L. 142: Use the plural form “sites” as you have several sampling points.

**Response:** We have been corrected it (L. 148)

L.145: As the selection of predictors is not the only step affecting the results of ENMs, consider replacing “determines” with “contributes to determine”.

**Response:** It has been replaced with “contribute to determine” (L. 151).

L. 147: “as the environmental data” is not very clear in this context. You could replace it with something “to individuate the variables being most informative for predicting…”.

**Response:** We have replaced “not only data availability but also biological relevance and previous studies should be considered as the environmental data for predicting the distribution of species” with “the selection of environmental variables should take into account not only data availability but also biological correlation” (L. 152).

L. 153: Replace “World Climate Database” with “WorldClim database”.

**Response:** We have been corrected it (L. 159).

L. 157: I suggest replacing the link you provide for the Harmonized World Soil Database with this latter https://www.fao.org/soils-portal/data-hub/soil-maps-and-databases/harmonized-world-soil-database-v12/en which directly leads to the database portal.

**Response:** We have been replaced it (L. 163-164)

L. 158: For more conciseness, consider replacing “from elevation variables using the spatial analysis function of ArcGIS (ESRI, Redlands, CA, USA), and the elevation data were obtained from WorldClim” with “from elevation data obtained from WorldClim, using the spatial analyst tool of ArcGIS software (ESRI, Redlands, CA, USA)”.

**Response:** We have been replaced it (L. 165-166)

L. 161: Consider replacing “due to the multicollinearity of environmental variables” with “in case of multicollinearity among the environmental variables”.

**Response:** We have replaced the whole sentence with “The multicollinearity among environmental variables will increase the uncertainty of model results.” (L.172-173).

L. 163-164: To avoid redundancy in this sentence, you may delete “to screen the environmental variables by group (Guo et al., 2021). We used r”

**Response:** We have been deleted it (L. 174).

Caption of Table 1: add something like “, along with the respective relative contribution scores from the fitted Maxent model” at the end of the sentence.

**Response:** The title of Table 1 was changed as “Environmental variables used or not used in model, along with the respective relative contribution scores from the fitted Maxent model”

Caption of Figure 2: Please report in the caption the extended forms of the abbreviations used in the diagram. I saw you reported them in the Methods subsection entitled “Comprehensive geospatial quality model”, yet the reader should be able to understand the Figure even without referring to the main text. Moreover, in the diagram of Figure 2 you should correct “envionmental” to “environmental”, and you may remove “result” after “habitat suitability” and “materials quality”.

**Response:** We have corrected the Figure 2.

**Fig. 2.** Schematic representation of the methodological approaches.

L. 179: You should add some reference after “one of the most successful SDMs in predicting species distribution”, for instance Norberg, A., Abrego, N., Blanchet, F. G., Adler, F. R., Anderson, B. J., Anttila, J., ... & Ovaskainen, O. (2019). A comprehensive evaluation of predictive performance of 33 species distribution models at species and community levels. Ecological monographs, 89(3), e01370.

**Response:** We have added the reference in the sentence “MaxEnt, one of the most successful SDMs in predicting species distribution (Norberg et al., 2019), is based on the maximum entropy principle and Bayesian estimates (Guo et al., 2018).”

**Reference:**
GUO Y, LI X, ZHAO Z, et al. 2018. Modeling the distribution of Populus euphratica in the Heihe River Basin, an inland river basin in an arid region of China. Science China-Earth Sciences [J], 61: 1669-1684. http://doi.org/10.1007/s11430-017-9241-2.
NORBERG A, ABREGO N, BLANCHET F G, et al. 2019. A comprehensive evaluation of predictive performance of 33 species distribution models at species and community levels. Ecological Monographs [J], 89. http://doi.org/10.1002/ecm.1370.

L. 181: Consider replacing “to project the distribution of E. sinica” with “to project the potential distribution of E. sinica across Inner Mongolia”. Moreover, to avoid redundancy, consider replacing “For the location point data, 75% of the data were selected as a training model, and the remaining…” with “75% of the occurrence data were selected to fit the model, and the remaining…”.

**Response:** We have changed “to project the distribution of E. sinica” with “to project the potential distribution of E. sinica across Inner Mongolia” (L. 192-194). And changed “For the location point data, 75% of the data were selected as a training model, and the remaining…” with “75% of the occurrence data were randomly selected to fit the model, and the remaining…” (L. 198)

L. 185: When at the beginning of a period, use the extended binomial name Ephedra sinica.

**Response:** we have corrected it (L. 210).

L. 188: AUC values can be also lower than 0.5, please correct.

**Response:** Thanks for suggestion. We have corrected the description into “AUC values range from 0 to 1…” (L. 214).

L. 189: I would say “a value of 0.5 indicates that model predictions are not better than random ones”, rather than” a value of 0.5 indicates that a model result is random”.

**Response:** We have changed the description into “when it below 0.5 indicating that model predictions are not better than random ones and 1 representing perfect discrimination.” (L. 215-216)

L.193: Consider “potential species distribution map was grouped into four classes” with “predicted suitability values were grouped into four classes”.

**Response:** We have replaced it (L. 219-220).

L. 219: “ is the result of the two ephedrine-type alkaloids in E. sinica in the th evaluation unit” is not very clear, consider replacing with “ is the output of the comprehensive geospatial quality model for the two ephedrine-type alkaloids of E. sinica in the th evaluation unit”.

**Response:** We have replaced it (L. 248-249).

L. 224: Consider using this formulation “the Maxent-based species distribution model (SDM) and the GLM-based geospatial quality model”.

**Response:** We have changed the whole sentence with “The MaxEnt-based species distribution model (SDM) and GLM-based geospatial quality model (GQM) served as the foundation for the comprehensive geospatial quality model (CGQM)” (L. 253-254).

L. 230-231: remove the dots within the extent measures of the ‘High’, ‘Medium’ and ‘Low’ suitability areas, as they may seem representing the decimal separator.

**Response:** We have corrected it, and change it with “The predicted suitability was divided into four levels: ‘High’ suitability (> 0.55) was 16.14 × 104 km^2^, ‘Medium’ suitability (0.34-0.56) was 26.04 × 104 km^2^, ‘Low’ suitability (0.14-0.34) was 28.57 × 104 km^2^, and the remaining area was ‘Unsuitable’ (<0.13) (Fig. 3).” (L. 258-261).

L. 242: bio15 is the coefficient of variation of precipitation, please correct. By the way, since bio15 is a ratio, it as no associated units of measurements so you should remove the “(mm)” indication in Table 1 while you can indicate “(%)”.

**Response:** We have corrected it (L. 271), and corrected the unit of bio15 in the table 1.

L. 244-246: “A variable response curve shows how each environmental variable affects a MaxEnt prediction and logistic predictions (Li et al., 2020). Hence, we used the response curve to obtain the parameters of the main influencing factors (Fig. 4)” is a bit weird sentence…consider replacing with something like “partial response curves reported in Fig. 4 show how Maxent predictions of habitat suitability for E. sinica changed along the gradient of the most influencing variables”.

**Response:** We have corrected it (L. 273-275).

L. 249: Here again, use % as “unit of measurement” for bio15.

**Response:** We have changed the whole sentence with “bio15 was more than 110.63%” (L. 277).

L. 246-250: You should specific here which was the threshold used to identify the suitable range along the partial response curve. Was it the lower end of the “Medium” suitability interval? Please clarify.

**Response:** We have changed the whole sentence (L. 275-280).

**Revision:** ”our model results indicated that the suitable range for bio7 was approximately 43.99 °C to 50.68 °C, and its optimal value was approximately 44 °C. In addition, ter ranged from 0.38 to 1, and its optimal value was 1～1.9 ; bio15 was more than 110.63%; srad ranged from 14776.56 kJ·m-2·d-1 to 16678.76 kJ·m-2·d-1; bio12 ranged from 195.89 mm to 477.35 mm, and its optimal value was 207.15 mm. Such a suitable range of environmental variables was more common in the ISH area.”

L. 255-256: These lines should be moved to the Methods section describing the GLM used to spatially model E and PE content. In that section, you should also specify that the response variables of the model were the content of E and PE.

**Response:** The lines has been moved to the methods section (L. 231-233). And changed the sentence into “We used the geospatial quality model to predict the geospatial pattern of E and PE. The relationship between E content and the environmental…” (L. 285-286).

L. 256: add a reference for the SPSS software, or at least the version used.

**Response:** we have corrected it (L. 232-233).

**Revision:** “We used stepwise regression to analyze the relationship between two ephedrine-type alkaloids and environmental variables with **SPSS (IBM SPSS Statistics 26; https://www.ibm.com/cn-zh/analytics/spss-statistics-software)** and constructed a spatial pattern model for E and PE (Guo et al., 2021).”

L. 263-264: To avoid redundancy in this sentence, you may simply state “The spatial distribution of the E content in the different regions, modelled as a function of bio2 and aspect, is shown in Fig. 5a”.

**Response:** We have corrected it (L. 291-292).

L. 268: “The geospatial quality model of PE content and the environmental variables was…” is a bit weird sentence… consider replacing with “The geospatial quality model of PE content was represented by the equation…”.

**Response:** We have replaced it (L. 296-297).

L. 271-272: This is an “obvious” repetition of L. 260-262…please take better care of English syntax and avoid repeating entire sentences.

**Response:** We have been replaced it (L. 298-301).

**Revision:** “This result demonstrated that the model had a significant effect, and the result is reliable. Bio8 is the most important variable for PE content, and the spatial quality model result of PE in the study area shown in Fig. 6a.”

L. 273-274: Same comment than for L. 268…try to merge the two sentences into one to avoid unnecessary repetitions.

**Response:** We have been changed it (L. 298-301).

**Revision:** “This result demonstrated that the model had a significant effect, and the result is reliable. Bio8 is the most important variable for PE content, and the spatial quality model result of PE in the study area shown in Fig. 6a.”

L. 278-283: As I commented above, the relationship between aspect variations and E content seems quite feeble…I would say that climate, and particularly temperature patterns, is apparently the main factor determining the distribution of E and PE content.

**Response:** We have changed it (L. 307-308).

**Revision:**” In summary, climate, and particularly temperature patterns, is apparently the main factor determining the accumulation of E and PE content.”

Figures 6 and 7: For better clarity, I suggest changing the legend titles to “E/PE content across Inner Mongolia” within the (a) panels and “E/PE content in important suitable habitats for E. sinica” within the (b) panels.

**Response:** We have corrected it. And we adjusted the order of the pictures, Fig.6-7 has been changed to Fig. 5-6.

**Revision:** “Fig. 5. a. E content across Inner Mongolia; b. E content in important suitable habitats for E. sinica” and “Fig. 6. a. PE content across Inner Mongolia; b. PE content in important suitable habitats for E. sinica.”

L.285: For better clarity, consider replacing “restricting the extent of two ephedrine-type alkaloids” with “to restrict the predictions of E and PE contents to areas with high suitability for E. sinica”.

**Response:** We have replaced it (L. 310).

**Revision:** “We used ISH as a constraint to restrict the predictions of E and PE contents to areas with high suitability for E. sinica.”

L. 286-288: To be clearer and more concise, consider replacing “According to the results of the CGQ model of E in E. sinica, the comprehensive spatial pattern was predicted. Here, we defined an E content greater than 0.8% as high quality, and E. sinica with high-quality E content was mainly distributed…” with something like “According to the CGQ model, areas with predicted high-quality E content (i.e. > 0.8%) were mainly distributed…”

**Response:** Thanks for suggestion. We have been changed it (L. 311).

**Revision:** “According to the CGQM, areas with high-quality E content (i.e.>0.8%) were found primarily in…”

L. 292: Replace “of ephedra” with “of “E. sinica”.

**Response:** We have replaced “of ephedra” with “of “E. sinica”. (L. 315).

L. 293-294: Here again, try to be more concise in your sentences. You can simply state “the predicted E content in important suitable areas for E. sinica was higher in the Horqin and Ulan Buh grasslands, while lower in the western Ordos grassland.”

**Response:** We have been corrected the description (L. 315-316).

**Revision:** “the predicted E content in ISH for E. sinica was higher in the Horqin grassland and Ulan Butong grassland, while lower in the western Ordos grassland.”

L. 295-297: Try to avoid these repetitions of entire sentences…You can simply state that “areas with high-quality PE content (i.e. > 0.6%) resulting from the CGQ model mainly spanned…”

**Response:** We have been corrected it into “Areas with high-quality PE content (i.e. > 0.8%) resulting from the CGQM mainly spanned in the Ordos grassland, Wulanchabu grassland and Ulan Butong grassland” (L. 317-318).

L. 305: If you know any previous work reporting these negative human impacts on E. sinica, put the corresponding reference here.

**Response:** We've added references to negative human influences here, i.e. (Abourashed et al. 2003; Ma et al. 2018) (L. 326).

**Reference added:**
Abourashed, E. A., A. T. El-Alfy, I. A. Khan, and L. Walker. 2003. 'Ephedra in perspective - a current review', Phytotherapy Research, 17: 703-12.
Ma, X., L. Fan, F. Mao, Y. Zhao, Y. Yan, H. Tian, R. Xu, Y. Peng, and H. Sui. 2018. 'Discrimination of three Ephedra species and their geographical origins based on multi-element fingerprinting by inductively coupled plasma mass spectrometry', Sci Rep, 8: 10271.

L. 309: the sentence “to predict geospatial pattern of two ephedrine-type alkaloids” is not very clear. You may change it with something like “to predict, across Inner Mongolia, the content of two ephedrine-type alkaloids characterising E. sinica”, here and throughout the manuscript.

**Response:** We have been corrected the description into “to predict, across Inner Mongolia, the content of two ephedrine-type alkaloids characterizing *E. sinica*” throughout the manuscript. (L. 194, 330).

L. 319-320: To avoid unnecessary repetitions, I suggest changing this sentence into “…various ecological factors, primarily climate, topography and soil properties”.

**Response:** We changing the sentence into” The growth and distribution of Ephedra and protection of phedrine-type alkaloids are the result of a complex interplay of various ecological factors, primarily climate, topography, soil and human properties.” (L. 339-340).

L. 323: Replace “bioclimate” with “bioclimatic”.

**Response:** We have corrected it (L. 345).

L. 326: which “cooccurring species”? please clarify.

**Response:** The cooccurring species is *Rose arabica* Crep. We’ve added it into Line 348.

L. 333: For a more appropriate English syntax, replace “have been buried in the soil for many years” with “persist in the soil for many years”.

**Response:** We have corrected it (L. 353).

**Revision:** “Because E. sinica is a perennial plant whose perennial roots persisting in the soil for many years.”

L.373: Consider replacing “a sporadic distribution” with either “a fragmented distribution” or “sporadic occurrences”.

**Response:** We have been replaced “from having 373 a widespread distribution to a sporadic distribution” with “from widespread to fragmented” (L. 400).

L. 384: You may delete “showed a trend that”.

**Response:** The sentence “showed a trend that” has been deleted (L. 429).

L. 387: Consider replacing “such as the high E content” with “exemplified by the high E content”.

**Response:** The “such as the high E content” has been replaced with “exemplified by the high E content” (L. 431-432).

L. 388: Add “low” after “the E contents were”.

**Response:** We have been corrected it (L. 433).

L. 425: For better clarity, consider replacing “were predicted in our results” with “were individuated through the fitted Maxent model”.

**Response:** We have been corrected the description into “were individuated through the fitted Maxent model” (L. 467).

References:

Cerasoli, F, Besnard, A, Marchand, M-A, D'Alessandro, P, Iannella, M, Biondi, M. (2021) Determinants of habitat suitability models transferability across geographically disjunct populations: Insights from Vipera ursinii ursinii. Ecology and Evolution; 11: 3991– 4011. https://doi.org/10.1002/ece3.7294

Cerasoli F, D’Alessandro P, Biondi M. (2022) Fine-Tuned Ecological Niche Models Unveil Climatic Suitability and Association with Vegetation Groups for Selected Chaetocnema Species in South Africa (Coleoptera: Chrysomelidae). Diversity; 14(2):100. https://doi.org/10.3390/d14020100

Hirzel, A. H., Le Lay, G., Helfer, V., Randin, C., & Guisan, A. (2006). Evaluating the ability of habitat suitability models to predict species presences. Ecological Modelling, 199(2), 142–152.
https://doi. org/10.1016/j.ecolm odel.2006.05.017

Jiménez-Valverde, A., Acevedo, P., Barbosa, A. M., Lobo, J. M., & Real, R. (2013). Discrimination capacity in species distribution models depends on the representativeness of the environmental domain. Global Ecology and Biogeography, 22(4), 508–516.https://doi.org/10.1111/geb.12007

Kass, J. M., Muscarella, R., Galante, P. J., Bohl, C. L., Pinilla‐Buitrago, G. E., Boria, R. A., ... & Anderson, R. P. (2022) ENMeval 2.0: Redesigned for customizable and reproducible modeling of species’ niches and distributions. Methods in Ecology and Evolution, 12(9)

Roberts, D. R., Bahn, V., Ciuti, S., Boyce, M. S., Elith, J., Guillera-Arroita, G., Hauenstein, S., Lahoz-Monfort, J. J., Schröder, B., Thuiller, W., Warton, D. I., Wintle, B. A., Hartig, F., & Dormann, C. F. (2017). Cross-validation strategies for data with temporal, spatial, hierarchical, or phylogenetic structure. Ecography, 40(8), 913–929.https://doi.org/10.1111/ecog.02881

Reviewer #3: it is interesting to predicte the comprehensive geospatial pattern of secondary metabolites in plants of a certain species. this article is an good case study in this area. however, i suppose the authors may do something to help the readeers to better understand the work.

**Response:** We thank Reviewer 3 for his/her thorough review, positive feedback, and constructive comments.

1. to add some background and case studies about the comprehensive quality model to determine the geospatial distribution of secondary metabolites in plants of a certain species.

**Response:** Thanks for the suggestion. We have been added the description of comprehensive quality model (L. 78-93).

**Revision:** “**Areas hosting suitable conditions for high-quality populations of a pharmacological plant species (i.e. populations whose individuals show high content of the plant’s medicinal components) may not completely match areas where the species actually occurs.** Thus, studies on high-quality suitable habitat for TCM plants should pay attention not only the suitable habitat but also the geospatial pattern of the effective medicinal component contents. We used a comprehensive quality model to determine the geospatial distribution of high-quality E. sinica. Li et al. (2020) predicted the distribution of three Coptis herbs (Coptis chinensis Franch., Coptis deltoidea C. Y. Cheng et Hsiao and Coptis teeta Wall) by using MaxEnt model and analyzed their chemical compositions by high-performance liquid chromatography (HPLC). They found that climate had a significant impact on the suitable habitat and alkaloid contents in three Coptis herbs. Guo et al. (2021) separately built a comprehensive habitat suitability model to identify suitable habitats and a spatial plant quality model to map the adenosine content pattern in Ophiocordyceps sinensis, and based on two models they developed a comprehensive niche modeling approach to predict the spatial pattern of O. sinensis quality. The study also looked at how environmental factors affected the distribution and adenosine content pattern of O. sinensis. **Jiang et al. (2022) used the MaxEnt to predict distribution of Scutellaria baicalensis Georgi, and a fuzzy matter element model was used to develop the relationship between environmental variables and baicalin content. Based on two models’ results, the habitat quality of the baicalin content in S. baicalensis across China were assessed, and they found that climatic factors wouldn’t only be an indicator for identifying the potential distribution area, but also for forming some secondary metabolites such as baicalin. However, there is currently no such spatial pattern analysis for Ephedra sinica Stapf.**”

**Reference:**
GUO Y, ZHAO Z, LI X 2021. Moderate warming will expand the suitable habitat of Ophiocordyceps sinensis and expand the area of O. sinensis with high adenosine content. Science of the Total Environment [J], 787. http://doi.org/10.1016/j.scitotenv.2021.147605.
LI J, FAN G, HE Y 2020. Predicting the current and future distribution of three Coptis herbs in China under climate change conditions, using the MaxEnt model and chemical analysis. Science of the Total Environment [J], 698. http://doi.org/10.1016/j.scitotenv.2019.134141.
JIANG Y, ZHAO Z, XIAO Y, et al. 2022. Identifying the habitat quality of Scutellaria baicalensis based on baicalin content using the fuzzy matter element model. Ecological Indicators [J], 141. http://doi.org/10.1016/j.ecolind.2022.109033.

2. to add the original data of E and PE content in E. sinica

**Response:** Sorry for the lack of original data of E and PE content, we will upload it as Supplementary Materials.

3. to discuss more about how enviromental factors effect the E and PE content in E. sinica.

**Response:** Thanks for the suggestion. We have been added the description of it (L. 376-391).

**Revision:** “Temperature is an important factor affecting the accumulation of alkaloids, making them show different trends. To cope with heat stress, plants implement various mechanisms, which is regulated at the molecular level, and potential genetic strategies to improve plant heat-stress tolerance enable plants to thrive under heat stress. Some scholars have found that peramine and Lolitrem B in perennial ryegrass will reach the maximum accumulation under high temperature in summer (Eerens et al., 1998). Furthermore, different alkaloids will exhibit different change trends when subjected to different temperature treatments (Sallam et al., 1977), the concentrations of ergine, ergonovine, and total ergot alkaloids were significantly higher when Festuca sinensis under short-term cold stress (Zhou et al., 2015). In our study, mean diurnal temperature was the key variable for the generation of E, and it is positively correlated with the change of E content. When the temperature rises, E content will increase. The most important factor for the production of PE was the mean temperature of the wettest quarter, and its change causes the reverse change of PE, that is, when it decreases, PE increases. Temperature influences photosynthesis, respiration, water relations and membrane stability, as well as hormone, primary and secondary metabolites levels in E. sinica (Wahid et al., 2007). Simultaneously, ephedrine-type alkaloids are important organic osmotic regulators, and precipitation will have a crucial impact on their production (Hsiao et al., 1976).”

**Reference:**
EERENS J P J, LUCAS R J, EASTON S, et al. 1998. Influence of the endophyte (Neotyphodium lolii) on morphology, physiology, and alkaloid synthesis of perennial ryegrass during high temperature and water stress. New Zealand Journal of Agricultural Research [J], 41: 219-226.
SALLAM L A R, NAIM N, ELREFAI A H 1977. THIN-LAYER CHROMATOGRAPHY OF SOME ERGOT ALKALOIDS. Fresenius Zeitschrift Fur Analytische Chemie [J], 284: 47-48.
ZHOU L, LI C, ZHANG X, et al. 2015. Effects of cold shocked Epichloe infected Festuca sinensis on ergot alkaloid accumulation. Fungal Ecology [J], 14: 99-104.
WAHID A, GELANI S, ASHRAF M, et al. 2007. Heat tolerance in plants: An overview. Environmental and Experimental Botany [J], 61: 199-223. http://doi.org/10.1016/j.envexpbot.2007.05.011.
HSIAO T C, ACEVEDO E, FERERES E, et al. 1976. STRESS METABOLISM - WATER STRESS, GROWTH, AND OSMOTIC ADJUSTMENT. Philosophical Transactions of the Royal Society B-Biological Sciences [J], 273: 479-500. http://doi.org/10.1098/rstb.1976.0026.

4. to improve the writing. I can understand the writhing, but I cannot say it is good and profesional. for instance, in the title, the name of the plant genus should not be just one letter. and what the unit of E and PE content in Fig 6-7?

**Response:** Thanks for the careful review. We have been polished our manuscript and improved the writing. The title has been replaced with “Predicting the comprehensive geospatial pattern of two ephedrine-type alkaloids for Ephedra sinica in Inner Mongolia”, and Fig. 6-7 have been corrected, and we adjusted the order of the pictures. Fig. 6-7 has been changed to Fig. 5-6.

**Revision:**

Fig. 5. a. E content across Inner Mongolia; b. E content in important suitable habitats for E. sinica

Fig. 6. a. PE content across Inner Mongolia; b. PE content in important suitable habitats for E. sinica.
