# Supplementary material for: Predicting the comprehensive geospatial pattern of two ephedrine-type alkaloids for Ephedra sinica in Inner Mongolia
Source: PLoS One. 2023 Apr 21;18(4):e0283967. doi: 10.1371/journal.pone.0283967 (PMC10121062; doi:10.1371/journal.pone.0283967)
Supplement: Attachment — Submitted filename: Response to Reviewers.docx [file pone.0283967.s005.docx]

**Response Letter**

We thank Journal feedback and encouragement for revision. We thank Editor for his/her positive feedback and encouragement for revision. The financial disclosure has been changed and we have updated the statement in our cover letter.

We are very grateful to the reviewer for the thorough review, positive feedback, and constructive comments. We have conducted profound reflection and revised the manuscript, adjusted the logic of the article, and polished our manuscript through language editing services to correct grammar and grammatical errors. Changes have been made in the revised manuscript based on the comments provided. Point-by-point responses are listed as follows.

Abstract: the period starting from L. 27 has several syntactical and grammatical errors, and it is too detailed about the optimization of model parameters. Consider replacing it with something like: “First, we used the MaxEnt software to map suitable habitats for E. sinica in inner Mongolia, based on occurrence data and a set of variables related to climate, topography and human impact. MaxEnt parametrization was optimized by evaluating alternative combinations of feature classes and values of the regularization multiplier. Secondly, a geospatial quality model was fitted to relate E and PE content to the same environmental variables and to predict their spatial patterns across the study area. Outputs from the two models were finally combined to map areas predicted to host both suitable conditions for E. sinica and high alkaloid content. Our results indicate that high-quality E content of E. sinica was mainly distributed in the Horqin grassland…”

**Response:** Thanks for the careful review. We have been replaced it (L. 27-38).

**Revision:** “In this study, MaxEnt software was utilized to map suitable habitats for *E. sinica* in Inner Mongolia based on occurrence data and a set of variables related to climate, soil, topography and human impact. The model parametrization was optimized by evaluating alternative combinations of feature classes and values of the regularization multiplier. Second, a geospatial quality model was fitted to relate E and PE contents to the same environmental variables and to predict their spatial patterns across the study area. Outputs from the two models were finally coupled to map areas predicted to have both suitable conditions for *E. sinica* and high alkaloid content. Our results indicate that *E. sinica* with high-quality E content was mainly distributed in the Horqin, Ulan Butong and Wulanchabu grasslands. *E. sinica* with high-quality PE content was primarily found in the Ordos, Wulanchabu and Ulan Butong grasslands. This study provides scientific information for the protection and sustainable utilization of *E. sinica*. It can also help to control and prevent desertification in Inner Mongolia.”

L.20: To improve clarity, consider replacing “is a type of traditional Chinese medicine that has a high medicinal value and large demands.” with “is a shrubby plant widely used in traditional Chinese medicine due to high medicinal value and large demands”.

**Response:** Thanks for the careful review. We changed the whole sentence (L. 21-22).

**Revision:** “*Ephedra sinica* Stapf. is a shrubby plant widely used in traditional Chinese medicine due to its high level of medicinal value, thus, it is in high demand.”

L. 26: To improve clarity, consider adding “content” after “E and PE”.

**Response:** We have been added it (L. 31).

**Revision:** “Second, a geospatial quality model was fitted to relate E and PE contents to the same environmental variables and to predict their spatial patterns across the study area.”

L. 44: Please replace “relying” with “relied” in the sentence “and the majority of these studies relying on species distribution models”.

**Response:** We have deleted the sentence.

L. 45-47: I suggest reversing the construction of this sentence to improve clarity. For instance, you could write: “Various SDM algorithms, including CLIMEX, DOMAIN, genetic algorithm for rule set production (GARP), BIOMOD-2 and maximum entropy (MaxEnt), have been used to assess ecological requirements and predict habitat suitability for several medicinal plant species”.

**Response:** We have replaced it (L. 68-71).

**Revision:** “Various SDM algorithms, including CLIMEX, DOMAIN, the genetic algorithm for rule set production (GARP), BIOMOD-2 and maximum entropy (MaxEnt), have been used to assess ecological requirements and predict habitat suitability for medicinal plant species.”

L. 53: Please remove “the” before “MaxEnt”, here and throughout the manuscript unless you place words like “software”, “algorithm”, “model” after “MaxEnt”.

**Response:** We have removed “the” before “MaxEnt” throughout the manuscript (L. 76, 261).

L. 60: Please replace “influencing” with “influence”.

**Response:** We have replaced it (L. 83).

L. 66: Please add “on” before “the suitable habitat” and before “the geospatial pattern”.

**Response:** We have changed the whole sentence (L. 87-89).

**Revision:** “Thus, studies on high-quality suitable habitat for TCM plants should pay attention not only on the suitable habitat but also on the geospatial pattern of the effective medicinal components.”

L. 67-68: I suggest moving the phrase “We used a comprehensive quality model to determine the geospatial distribution of high-quality E. sinica” directly at the end of this paragraph (L.83), so that you start introducing the methods and scope of your study after having exemplified results from previous studies on medicinal plants. I also suggest adding “populations” after “E. sinica”.

**Response:** Thanks for the suggestion. But for the sake of the article as a whole, we deleted this paragraph.

L. 78: Please replace “develop” with “assess/estimate/analyze”.

**Response:** We have changed the whole sentence (L. 98-100).

**Revision:** “Furthermore, a study of habitat influence on the baicalin content in *Scutellaria baicalensis* Georgi revealed that climatic factors were important indicators of both the potential distribution and baicalin production of the plant.”

L. 80-82: “they found that climatic factors wouldn’t only be an indicator for identifying the potential distribution area, but also for forming some secondary metabolites such as baicalin” is a bit weird sentence…consider replacing it with something like “they found that climatic factors are useful indicators of both plants’ potential distribution and production of some secondary metabolites such as baicalin”.

**Response:** We have changed the whole sentence (L. 98-100).

**Revision:** “Furthermore, a study of habitat influence on the baicalin content in *Scutellaria baicalensis* Georgi revealed that climatic factors were important indicators of both the potential distribution and baicalin production of the plant.”

L. 86: Please replace the comma after “colds and obesity[20]” with a semicolon or a full stop.

**Response:** We have replaced it (L. 49).

**Revision:** “It is commonly used in TCM to treat a variety of ailments, including bronchitis, asthma, whooping cough, colds and obesity[5]. ”

L. 100: Please replace “overharvested” with “overharvesting”.

**Response:** We have replaced it (L. 63).

**Revision:** “However, the increasing international market demand for natural alkaloid contents has led to **overharvesting** of *E. sinica.*”

L. 105: Add “geospatial” before “patterns” and add “these” before “two”.

**Response:** We have changed the sentence (L. 104).

**Revision:** “To generate and accumulate effective components, the plant must be alive; thus, when simulating the geospatial patterns of these two ephedrine-type alkaloids, habitat suitability for *E. sinica* should be delimited first.”

L. 108: Consider replacing “the spatial pattern of two ephedrine-type alkaloids” with “the spatial patterns of E and PE content”.

**Response:** We have changed the sentence (L. 107-108).

**Revision:** “Then we used a geospatial quality model and ephedrine-type alkaloid content data to predict **the spatial patterns of E and PE contents**.”

L. 110: To improve clarity, consider replacing “habitat distribution and effective components” with “the distribution of suitable habitats for E. sinica and effective production of ephedrine-type alkaloids”.

**Response:** We have changed it (L. 110-111).

**Revision:** “The results will help determine which environmental factors influence **the distribution of suitable habitats for *E. sinica* and the effective production of ephedrine-type alkaloids**.”

L. 122: To improve syntactical correctness, consider replacing “…Ordos. While the western region is arid, with the Alxa desert.” with “…Ordos, while the western region is arid and includes the Alxa desert”.

**Response:** We have changed it (L. 121-122).

**Revision:** “The grasslands are mostly distributed in eastern and central Inner Mongolia, including Hulun Buir, Uragai, Xilingol, Horqin, Ulan Butong, Wulanchabu and **Ordos, while the western region is arid and includes the Alxa desert**.”

L. 123: To improve syntactical correctness and clearness, consider replacing “…and sample sites with two ephedrine-type alkaloids were obtained through published researchers” with “…and sample sites with content data for the two target two ephedrine-type alkaloids were obtained through published literature”.

**Response:** Thanks for the suggestion. We have replaced it (L. 123-124).

**Revision:** “Occurrence records of *E. sinica* **and sample sites with content data for the two target ephedrine-type alkaloids were obtained through published literature**.”

Table 1: Please replace “Isothermally” with “Isothermality”.

**Response:** We have been fixed the description of bio3.

**Revision:**

| **Table 1**  Environmental variables used or not used in model, along with the respective relative contribution scores from the fitted Maxent model | | | |  |
| --- | --- | --- | --- | --- |
| Variable type | Code(Unit) | Description | Variables used in modeling | Contribution (%) |
| Climatic variables | bio1(℃) | Annual mean air temperature | √ | 2.1 |
|  | bio2(℃) | Mean diurnal temperature range (max. temp – min. temp) | √ | 0.8 |
|  | bio3 | **Isothermality (Bio2/Bio7) × 100** | √ | 4.3 |
|  | bio4(℃) | Temperature seasonality |  |  |
|  | bio5(℃) | Max temperature of warmest month |  |  |
|  | bio6(℃) | Min temperature of coldest month |  |  |
|  | bio7(℃) | Temperature annual range | √ | 26.6 |
|  | bio8(℃) | Mean temperature of wettest quarter |  |  |
|  | bio9(℃) | Mean temperature of driest quarter |  |  |
|  | bio10(℃) | Mean temperature of warmest quarter |  |  |
|  | bio11(℃) | Mean temperature of coldest quarter |  |  |
|  | bio12(mm) | Annual precipitation | √ | 8.4 |
|  | bio13(mm) | Precipitation of wettest month |  |  |
|  | bio14(mm) | Precipitation of driest month |  |  |
|  | bio15(%) | Coefficient of variation of precipitation | √ | 19.3 |
|  | bio16(mm) | Precipitation of wettest quarter |  |  |
|  | bio17(mm) | Precipitation of the driest quarter |  |  |
|  | bio18(mm) | Precipitation of warmest quarter |  |  |
|  | bio19(mm) | Precipitation of coldest quarter |  |  |
|  | srad(kJ·m^-2^·d^-1^) | Solar radiation | √ | 10.9 |
|  | vapr(hPa) | Vapour pressure | √ | 0.6 |
| Soil variables | soil | Soil type | √ | 0.3 |
|  | clay1 | Topsoil Clay Fraction (0 - 30cm) |  |  |
|  | clay2 | Subsoil Clay Fraction (30 - 100cm) |  |  |
|  | sand1 | Topsoil Sand Fraction (0 - 30cm) | √ | 0.7 |
|  | sand2 | Subsoil Sand Fraction (30 - 100cm) |  |  |
|  | sq1 | Nutrient availability | √ | 0.3 |
|  | sq2 | Nutrient retention capacity |  |  |
|  | sq3 | Rooting conditions | √ | 1.1 |
|  | sq4 | Oxygen availability to roots |  |  |
|  | sq5 | Excess salts | √ | 1.1 |
|  | sq6 | Toxicity | √ | 0.2 |
|  | sq7 | Workability (constraining field management) |  |  |
| Topographical variables | ele(m) | Elevation above sea level | √ | 0.2 |
|  | slop(%) | Slope |  |  |
|  | asp(degrees) | Aspect | √ | 0.1 |
| Human activity variables | hf | Human Footprint |  |  |
|  | den | Population Density |  |  |
|  | ter | Global Human Modification of Terrestrial Systems | √ | 23 |

L.167-169: To improve clearness and readability, I would change the period structure to something like “By coupling a species distribution model with a geospatial quality model (GQM), we developed a comprehensive geospatial quality model (CGQM) to predict the geospatial pattern of two ephedrine-type alkaloids in E. sinica (Fig. 2).

**Response:** We have changed it (L. 167-169).

**Revision:** “By coupling a species distribution model with a geospatial quality model (GQM), we developed a comprehensive geospatial quality model (CGQM) to predict the geospatial pattern of two ephedrine-type alkaloids in *E. sinica* (Fig. 2).”

Figure 2: “Envionment” instead of “Environmental” is still present in the “Geospatial quality model” panel; please fix it. Moreover, consider adding “implemented” before “methodological approaches” in the caption. Finally, to improve clearness of the Figure content, I also suggest:

- replacing “Spatial distribution of medicinal quality materials (GQM)” with “areas with high predicted alkaloid content”, also modifying the heading to “Geospatial Quality Model (GQM)”.

- replacing “High quality suitable habitat of major content” with “highly suitable areas with high predicted alkaloid content” in the “Comprehensive Geospatial Quality Model (CGQM)” panel.

**Response:** We have corrected the Figure 2.

**Revision:**

**Fig. 2.** Schematic representation of the methodological approaches.

L. 185: when writing “omission rates (E = 0.05)”, did you mean that you selected 0.05 as threshold value for omission rate thus considering only those models that did not exceed this value? Please clarify.

**Response:** We have changed it (L. 184).

**Revision:** “Model selection was based on **(i) omission rates (E ≤ 0.05)**, which represent the proportion of incorrectly predicted test records by the model, and (ii) Delta Akaike Information Criterion (dAIC < 2), which indicates the models with the best trade-offs between data fitting and complexity.”

L. 189-191: This paragraph is poorly written. Consider adjusting to something like “Finally, the selected MaxEnt model was fitted using linear and threshold features (i.e. FC = lt), with RM = 3.5. The maximum number of iterations was set to 1000, logistic output format was set to logistic, and 10 replicated runs of cross-validation were used to reduce the uncertainty of the model.”

**Response:** We have changed it (L. 188-192).

**Revision:** “Finally, the selected MaxEnt model was fitted using linear and threshold features (i.e., FC = lt), with RM = 3.5. The maximum number of iterations was set to 1000, the logistic output format was set to logistic, and 10 replicated runs of cross-validation were used to reduce the uncertainty of the model.”

L. 195: Add the final “l” to “mode

**Response:** We have corrected it (L. 197).

L. 196: Please change “when it below 0.5 indicating…” to “with values lower than 0.5 indicating…”

**Response:** We have changed it (L. 198).

**Revision:** “AUC values range from 0 to 1, **with values lower than 0.5 indicating** that model predictions are not better than random ones and 1 representing perfect discrimination.”

L. 200-201: Consider moving this sentence about the split of predicted suitability into discrete classes to L. 193, so that it would be better connected to the description of MaxEnt output.

**Response:** We have moved it (L. 194-195).

**Revision:** “The final predicted suitability values were grouped via natural breaks into four classes of High, Medium, Low and Unsuitable.”

L.212-213: As in the previous version you stated that stepwise regression was used for model refinement (i.e. variable selection), consider changing this part to something like “We used SPSS (IBM SPSS Statistics 26; https://www.ibm.com/cn-zh/analytics/spss-statistics-software) to perform a stepwise regression for refining the initial GLM, thus maintaining only the informative predictors”.

**Response:** We have changed the sentence (L. 213-215).

**Revision:** “We used SPSS (IBM SPSS Statistics 26; https://www.ibm.com/cn-zh/analytics/spss-statistics-software) to perform a stepwise regression to refine the initial GLM, thus maintaining only the informative predictors.”

L. 234: Following my previous comment, change “GLM-based” with “regression-based”.

**Response:** We have changed the sentence (L. 239).

**Revision:** “The MaxEnt-based species distribution model (SDM) and regression-based geospatial quality model served as the foundation for the comprehensive geospatial quality model.”

L. 238-239: As the AIC value is not very informative by itself, you may remove it and change the sentence to “and AUC value of 0.960, indicating that the model performed well and was reliable in discriminating suitable areas for E. sinica”.

**Response:** We have changed it (L. 243-245).

**Revision:** “and AUC value of 0.965, indicating that the model performed well and was reliable in discriminating suitable areas for *E. sinica*.”

L.240-245: I suggest you to change “was” with “covered” when writing about the extent the different suitability classes spanned. Moreover, in explicating the extent of the different categories, avoid using XXX ˟ YYY km2 : you should either use the form XXX ˟ YYY km or directly enumerate the overall extent, such as ZZZ km2 . Please also change “‘High’ suitable habitat mainly distributed in…” with “highly suitable habitat was mainly distributed in…”.

**Response:** We have changed them (L. 245-248).

**Revision:** “The predicted suitability was divided into four levels: high suitability (> 0.55) was **161400 km^2^**, medium suitability (0.34-0.56) was **260400 km^2^**, low suitability (0.14-0.34) was **285700 km^2^**, and the remaining area was unsuitable (<0.13) (Fig. 3). **High-suitability habitat** was mainly distributed in the Horqin, Wulanchabu and Ordos grasslands.”

L. 250-254: Here you state that the most influential variable was bio7, but Fig. 4a shows the partial response curve for bio17…please fix this.

**Response:** We apologize for the mistake and fixed it (L. 245-248).

**Revision:**

**Fig. 4.** Response curves for important environmental predictors in the species distribution model for *E. sinica.*

L. 256: You should not start a sentence with “And” after a full stop. Use instead something like “Further”, “Moreover”, “Additionally”.

**Response:** We changed “And” with “Moreover” (L. 261).

**Revision:** “**Moreover,** our model results indicated that the suitable range for bio7...”

L. 257-260: I don’t think it is very informative to specify with such precision the range of values the partial response curves span. One reason is that this range could be influenced by the clamping procedure used within the MaxEnt software to extend the response curves beyond the range of the training values…for instance, how could the optimal value for ter be “1～1.9” if you state that it “ranged from 0.38 to 1”. I suggest you focus instead on the trends resulting from the curves (e.g. increasing suitability for E. sinica with higher values of ter and bio15) and on the range of values (not the precise values which are difficult to assess from the curves) corresponding to the highest predicted suitability and/or to abrupt changes in the trend of the focal curve.

**Response:** We changed the sentence (L. 261-267).

**Revision:** “Moreover, our model results indicated that the suitable range for bio7 was approximately 45 °C to 50 °C, and the optimal value of bio7 was approximately 46 °C. In addition, bio15 ranged from 105% to 130% with an optimal value at approximately 115%; srad ranged from 15000 kJ·m-2·d-1 to 16500 kJ·m-2·d-1, with the highest predicted suitability value at 15500 kJ·m-2·d-1; bio 12 ranged from 200 mm to 450 mm with an optimal elevation at approximately 270 mm. Furthermore, as the value of ter increased above 0.1, an increase in the probability of presence was observed.”

L. 270: Please change “This result showed that the model had a remarkable effect,” with something like “This result suggests that the model explained a not negligible portion of the spatial variability in E content”. A model has no effect…the model you used just estimate the portion of variability in the response variable which could be causally related to the retained predictors…

**Response:** We changed the sentence (L. 277-278).

**Revision:** “This result suggests that the model explained a nonnegligible portion of the spatial variability in E content,”

L. 282: As for the previous comment, please replace “the model had a significant effect, and the result is reliable” with something like “the model has a noticeable explanatory power”.

**Response:** We changed the sentence (L. 285-286).

**Revision:** “This result demonstrated that the model has noticeable explanatory power.”

L. 283: To improve clearness and syntactical correctness, please replace “and the spatial quality model result of PE in the study area shown in…” with something like “the projection of PE content across Inner Mongolia resulting from the corresponding geospatial quality model is shown in…”

**Response:** We changed the sentence (L. 287-288).

**Revision:** “the projection of PE content across Inner Mongolia resulting from the corresponding geospatial quality model is shown in Fig. 6a.”

L. 290-293: The wording here is a bit blurry…consider replacing with something like “Climate, and particularly temperature patterns, is apparently the main factor determining the accumulation of E and PE in E. sinica samples because bio2 and bio8 emerged as the main variables influencing the spatial distribution of E and PE content”.

**Response:** We changed the sentence (L. 289-291).

**Revision:** “Climate, particularly temperature patterns, is apparently the main factor determining the accumulation of E and PE contents in *E. sinica* samples because bio2 and bio8 emerged as the main variables influencing the spatial distribution of E and PE contents.”

L. 298: To improve clearness and syntactical correctness, please replace “The high-quality E content in ISH was approximately 13.86 × 104 km2” with something like “the extent of ISH areas predicted to host high-quality E was approximately ZZZ km2 ”.

**Response:** We changed it (L. 296).

**Revision:** “and they covered approximately **138600 km**^2^.”

L. 314-315: Consider replacing “and the AUC value was 0.960, indicating excellent performance in evaluating the accuracy of the results. Further, we used a GLM to…” with something like “and the AUC value was 0.960, indicating excellent discrimination performance. Further, we used a stepwise regression-based model to…”

**Response:** We changed the sentence (L. 312-313).

**Revision:** “This study used a MaxEnt model to simulate the potential distribution of *E. sinica* in Inner Mongolia, **for which the AUC value was 0.960, indicating excellent discrimination performance. Furthermore, we used a stepwise regression-based model to** predict, across Inner Mongolia…”

L. 318: Add “these” before “two models”.

**Response:** We added it (L. 316).

**Revision:** “We obtained the final comprehensive geospatial distribution for two ephedrine-type alkaloids of *E. sinica* by coupling the results of **these two models.**”

L. 320-321: You should better explicit how your results would help to “promote the sustainable use and appropriate conservation of E. sinica in Inner Mongolia”, for instance adding something like “by indicating which areas potentially host higher E. sinica populations with ephedrine-type alkaloid content so as to make its harvesting more efficient”. If you do this, you could then start the following sentence with “Our results will also make an important contribution to the enhancement of medicinal value and protection of biodiversity in Inner Mongolia”.

**Response:** We added it (L. 318-321).

**Revision:** “These findings will promote the sustainable use and appropriate conservation of *E. sinica* in Inner Mongolia **by indicating which areas can potentially host larger *E. sinica* populations with high-quality ephedrine-type alkaloid content; these conditions can increase harvesting efficiency. Our results also make an important contribution to the enhancement of medicinal value and protection of biodiversity in Inner Mongolia.**”

L. 326-327: Please remove this sentence as it is totally redundant with the previous one.

**Response:** We removed it.

L. 333-334: The wording here is a bit awkward; consider changing to something like: “In addition, climate emerged as the main factor driving the distribution of Rosa arabica Crep. which frequently cooccurs with Ephedra species”.

**Response:** We changed the sentence (L. 350-351).

**Revision:** “In addition, climate emerged as the main factor driving the distribution of *Rosa arabica* Crep., which frequently cooccurs with Ephedra species”

L. 335: Please add “for E. sinica” after “habitat suitability”.

**Response:** We changed the sentence (L. 353).

**Revision:** “Similar to previous studies, our results indicated that climate-related predictors were the main variables affecting habitat suitability **for *E. sinica.***”

L. 339: “The temperature factor provides E. sinica an comfortable environment to grow up…” is a bit awkward sentence; consider replacing with something like “Annual temperature range contributes to determine the degree to which E. sinica populations of Inner Mongolia experience comfortable thermal conditions to grow up…”

**Response:** We changed the sentence (L. 357-359).

**Revision:** “Annual temperature range helps determine the appropriate thermal conditions that *E. sinica* populations in Inner Mongolia need to grow,”

L. 340: Please remove the word “factor” after precipitation…unless you’re referring to a specific precipitation-related variable it is uninformative.

**Response:** We changed the sentence (L. 366).

L. 347: To avoid redundancy, consider this formulation: “…for the distribution of E. sinica, because it grows…”

**Response:** We changed the sentence (L. 366).

**Revision:** “Furthermore, global human modification of terrestrial systems (23%) is an important variable **for the distribution of *E. sinica* because *E. sinica* grows** primarily in grasslands, where farming and animal husbandry activities and cultivation behavior change the land cover.”

L. 349-351: Here you state soil variables are also important, but this did not emerge from your MaxEnt model, so you should discuss why…for instance, you may hypothesize that soil properties are important drivers of E distribution at finer spatial scales than the regional scale of your research.

**Response:** We changed the sentence (L. 367-370).

**Revision:** “However, soil variables, such as soil texture, vertical soil properties and root infiltration conditions, directly affect the survival and growth of perennial roots, thus indicating that soil properties are important drivers of *E. sinica* distribution at finer spatial scales.”

L. 352: “Secondary metabolite from plants is the unique sources…” is not so convincing. Pharmaceuticals, flavours etc. also derive from animal or mineral material. Please replace with “Secondary metabolites from plants are among the main sources…”

**Response:** We changed the sentence (L. 371).

**Revision:** “**Secondary metabolites from plants are among the main sources** of pharmaceuticals, food additives, flavors, and other industrial materials.”

L. 357: Please correct to “Ephedrine-type alkaloids are the main secondary metabolites…”

**Response:** We changed the sentence (L. 376-378).

**Revision:** “**Ephedrine-type alkaloids, which are the main secondary metabolites** of Ephedra, are important for estimating the quality of crude drugs and safe medication.”

L. 360: Please correct to “various mechanisms, which are regulated…”

**Response:** We changed the sentence (L. 380-382).

**Revision:** “To cope with heat stress, plants implement **various mechanisms that are regulated** at the molecular level, which can improve plant heat stress tolerance and enable plants to thrive under heat stress.”

L. 361: You should expand a bit about these “genetic strategies” to make the concept clearer to a reader not experienced of plants physiological regulatory mechanisms.

**Response:** After reviewing the literature, we found that there was no professional term to explain “genetic strategies”, so we changed the sentence (L. 384-386).

**Revision:** “To cope with heat stress, plants implement various mechanisms that are regulated at the molecular level, which can improve plant heat stress tolerance and enable plants to thrive under heat stress.”

L. 365: Break the sentence here: “…temperature treatments [70]; for instance, the concentrations of ergine, ergonovine…”

**Response:** We changed the sentence (L. 384-385).

**Revision:** “Furthermore, different alkaloids exhibit different change trends when subjected to different **temperature treatments [71]; for instance, the concentrations of ergine,** ergonovine, and total ergot alkaloids are significantly higher when Festuca sinensis is maintained under short-term cold stress.”

L. 366: Please add something like “is maintained” or “is put” before “under short-term cold stress”.

**Response:** We added “is maintained” before “under short-term cold stress” (L. 386).

**Revision:** “and total ergot alkaloids are significantly higher when *Festuca sinensis* **is maintained** under short-term cold stress.”

L. 367: Add ‘range’ after ‘mean diurnal temperature’.

**Response:** We added it (L. 387).

**Revision:** “In our study, the mean diurnal temperature **range** was the key variable for the generation of ephedrine, and it was positively correlated with the change in E content”

L. 367-369: As here you’re citing for the first time in the Discussion the two ephedrine-type alkaloids, you may use their extended names to help the reader recall what you’re talking about, then continuing with their abbreviations in the subsequent paragraphs.

**Response:** We have been corrected it (L. 387,389).

**Revision:** “In our study, the mean diurnal temperature range was the key variable for the generation of **ephedrine (E)**, and it was positively correlated with the change in E content. When the temperature rose, the E content increased. The most important factor for the production of **pseudoephedrine (PE)** was the mean temperature of the wettest quarter, which was negatively correlated with PE content, so that the latter increased with lower temperature in the wettest season.”

L. 370: To ameliorate the wording, consider changing to something like “which was negatively correlated to PE content, so that the latter increases with lower temperature in the wettest season.”

**Response:** We have been changed the sentence (L. 390-391).

**Revision:** “The most important factor for the production of pseudoephedrine (PE) was the mean temperature of the wettest quarter, **which was negatively correlated with PE content, so that the latter increased with lower temperature in the wettest season.**”

L. 378: Please change ‘species’ to ‘populations/variety’ if you’re specifically referring to E. sinica.

**Response:** We have been changed ‘species’ to ‘populations’ (L. 398).

**Revision:** “The survival and competition potential of **local populations** of *E. sinica* should be evaluated carefully, and the destruction of local habitats and biodiversity should be avoided in the process of artificial cultivation”

L. 379: Consider changing “ecological environment” with “habitats”.

**Response:** We have been changed “ecological environment” with “habitats” (L. 399).

**Revision:** “The survival and competition potential of **local populations** of *E. sinica* should be evaluated carefully, and the destruction of local habitats and biodiversity should be avoided in the process of artificial cultivation”

L. 384-388: Here you write “E. sincia” instead of “E. sinica”; please correct.

**Response:** We apologize for the misspelling. We corrected it (L. 422-426).

**Revision:** “As a result of rapidly changing climate and human-caused ecological destruction, the distribution of ***E. sinica*** in Inner Mongolia has shifted from widespread to fragmented distribution in recent decades [78, 79]. Meanwhile, because of the high medicinal value of ***E. sinica***, which is widely used in both traditional Chinese medicine and Western medicine, existing ***E. sinica*** populations and potential suitable areas are critical for its future protection and reforestation. We propose the following conservation management strategies for ***E. sinica*** in Inner Mongolia based on our findings.”

L. 384: Replace “have been” with “has been” as you’re referring to a singular subject, “E. sinica”.

**Response:** We corrected it (L. 423).

**Revision:** “Meanwhile, because of the high medicinal value of *E. sinica*, **which is widely used** in both traditional Chinese medicine and Western medicine, existing *E. sinica* populations and potential suitable areas are critical for its future protection and reforestation. We propose the following conservation management strategies for *E. sinica* in Inner Mongolia based on our findings.”

L. 385: Add “populations” or “occurrence localities” after “existing E. sinica”.

**Response:** We added it (L. 425).

**Revision:** “Meanwhile, because of the high medicinal value of *E. sinica*, which is widely used in both traditional Chinese medicine and Western medicine, existing *E. sinica* **populations** and potential suitable areas are critical for its future protection and reforestation. We propose the following conservation management strategies for *E. sinica* in Inner Mongolia based on our findings.”

L. 391: To avoid repeating “conservation” so many times, consider replacing “to achieve species conservation in recent years” with “to preserve plant species”.

**Response:** We added it (L. 429-430).

**Revision:** “Conservation methods such as in situ conservation, near-field conservation, ex situ conservation, and germplasm conservation have been gradually applied **to preserve plant species**.”

L. 393-394: These two lines could be removed as you few lines later you specify the ISH areas which should be given restoration priority.

**Response:** We removed the sentence to L. 432-433.

**Revision:** “In situ protection should be strengthened in regions where wild *E. sinica* exists, and protection-relevant policies and measures to prohibit the destruction of wild *E. sinica* should be issued. **Furthermore, reasonable restoration project planning should be encouraged in the predicted potential suitable habitat areas.** The majority of important suitable habitat for *E. sinica* in our study was located in the Horqin, Wulanchabu, Ordos and Ulan Butong grasslands.”

L. 396: Consider removing the “ISH covered ca. 42.18 × 104 km2” part, because repeating in the Discussion quantitative details you already reported in the Results is not so useful.

**Response:** We removed the sentence (L. 434).

L. 399 and L. 405: You may remove the saxon genitive here as you never used it above when referring to E. sinica.

**Response:** We removed the sentence (L. 438 and L. 402).

L. 425: Please change “need” to “needed” as here you’re referring to what you’ve done for your specific research.

**Response:** We changed “need” to “needed” (L. 325).

**Revision:** “We **needed** to collect not only occurrence records of *E. sinica* but also sites with corresponding effective component data; however, collecting data on components is difficult because few studies have been done on the spatial patterns of the two ephedrine-type alkaloids.”

L. 429: To improve the wording, consider changing to “…the medicinal parts of the target plant species”.

**Response:** We changed the sentence (L. 329).

**Revision:** “We believe that component data richness could be increased by using field work to collect **the medicinal parts of the target plant species** and by implementing additional laboratory work to measure the effective components if corresponding hardware devices are available.”

L. 430: “in future studies” is a bit redundant here, you may remove it.

**Response:** We removed it (L. 331).

L. 433: To improve the wording, consider changing to “..environmental variables, plant distribution as well as metabolite contents”.

**Response:** We changed the sentence (L. 333-334).

**Revision:** “and we need to intuitively understand the relationship between **environmental variables, plant distribution and metabolite contents.**”

L. 435-439: To improve the wording, consider changing to “…According to the chosen evaluation metrics, both models performed well, and they identified climate, human modifications and, secondarily, topography as key environmental factors affecting the distribution of E. sinica and the spatial pattern of alkaloids content. The fact that we used environmental predictors with approximately 1 Km2 resolution may facilitate extracting general guidelines on conservation and management of E. sinica in Inner Mongolia at a relatively fine spatial scale”.

**Response:** We changed the sentence (L. 336-341).

**Revision:** “According to the chosen evaluation metrics, both models performed well, and they identified climate, human modifications and, secondarily, topography as key environmental factors affecting the distribution of *E. sinica* and the spatial pattern of alkaloid content. The fact that we used environmental predictors with approximately 1 km2 resolution may facilitate the development of general guidelines on the conservation and management of *E. sinica* in Inner Mongolia at a relatively fine spatial scale.”

L. 443: As for the start of the Discussion, I suggest using the extended name of E and PE here that you cite them for the first time in the Conclusions section.

**Response:** We corrected it (L. 443).

**Revision:** “To analyze the geospatial pattern of **ephedrine(E)** and **pseudoephedrine(PE)** contents in *E. sinica*…”

L. 448: Add human modifications as a key predictor, as it emerged clearly within your updated Maxent model.

**Response:** We corrected it (L. 448-449).

**Revision:** “Climate, topographic and **human activity** variables had the greatest influence on *E. sinica*.”

L. 449: Please replace “environmental variables” with “modelled habitat suitability”, as in its current form this sentence is unclear

**Response:** We replaced “environmental variables” with “modelled habitat suitability” (L. 450).

**Revision:** “The projected geospatial pattern and **modeled habitat suitability** for *E. sinica* are good references for sustainable use and conservation strategies.”
